# Cyclophosphamide-Induced Nephrotoxicity and Nephroprotection in Rodent Models: A Systematic Review and Random-Effects Meta-Analysis (2010–2025)

**DOI:** 10.3390/jox16020048

**Published:** 2026-03-04

**Authors:** Denis Oberiukhtin, Anton Chernitskiy, Desheng Hu, Alexey Sarapultsev

**Affiliations:** 1 Institute of Immunology and Physiology, Ural Branch of the Russian Academy of Science, 106 Pervomaiskaya Street, Ekaterinburg 620049, Russia; oberuhtindenis@gmail.com; 2 Ural Federal Agrarian Scientific Research Centre, Ural Branch of the Russian Academy of Sciences, 112A Ulitsa Belinskogo, Ekaterinburg 620061, Russia; cherae@mail.ru; 3 Department of Integrated Traditional Chinese and Western Medicine, Union Hospital, Tongji Medical College, Huazhong University of Science and Technology, No. 1277 Jiefang Avenue, Wuhan 430022, China; desheng.hu@hust.edu.cn; 4 Hubei Key Laboratory of Biological Targeted Therapy, Union Hospital, Tongji Medical College, Huazhong University of Science and Technology, No. 1277 Jiefang Avenue, Wuhan 430022, China; 5 China-Russia Medical Research Center for Stress Immunology, Union Hospital, Tongji Medical College, Huazhong University of Science and Technology, No. 1277 Jiefang Avenue, Wuhan 430022, China

**Keywords:** acute kidney injury, apoptosis, cyclophosphamide, inflammation, meta-analysis, nephroprotection, nephrotoxicity, oxidative stress, rodent models, systematic review

## Abstract

Cyclophosphamide (CP) is extensively used in oncology and as an immunosuppressant, but dose-limiting renal injury remains a major constraint. We systematically reviewed in vivo rodent models of CP nephrotoxicity (2010–2025) and meta-analysed core outcomes while separating the model effect (CP vs. control) from the treatment effect (intervention + CP vs. CP-only). Fifty-four studies met eligibility criteria, and random-effects syntheses were feasible for serum creatinine, serum urea, and renal oxidative stress markers. CP produced a marked functional deterioration, increasing serum creatinine by 1.059 mg/dL (95% CI 0.517–1.601; *k* = 9) and serum urea by 39.852 mg/dL (95% CI 6.557–73.148; *k* = 9). Across intervention studies, protective effects were most consistently expressed in oxidative endpoints (MDA/TBARS reduction and glutathione preservation), whereas functional recovery estimates were more variable and frequently limited by incomplete reporting and between-study heterogeneity. Overall, the evidence base supports CP as a robust preclinical model of combined functional and redox-mediated renal injury and indicates that multiple mechanistic classes of interventions can partially mitigate injury, but current reporting and design heterogeneity preclude reliable ranking of candidate agents. The protocol was registered on OSF.

## 1. Introduction

Cyclophosphamide (CP) remains a cornerstone alkylating agent in oncology and an established immunosuppressant in selected immune-mediated indications, yet its therapeutic use continues to be constrained by treatment-limiting toxicities that can disrupt dose intensity, continuity, and long-term outcomes [1]. Renal injury is a recurring concern in experimental CP toxicology and is clinically plausible in contexts where hydration status, comorbidity, co-medications, and inter-individual variation in bioactivation and clearance narrow renal reserve [1]. Consequently, a large amount of preclinical literature has accumulated in rodent models describing CP-induced renal dysfunction and tissue injury, alongside parallel literature claiming nephroprotection across diverse pharmacological and nutraceutical strategies.

CP is an oxazaphosphorine agent administered as an inactive prodrug that requires cytochrome P450-dependent biotransformation (with clinically relevant inter-individual variability) to 4-hydroxycyclophosphamide/aldophosphamide, followed by generation of phosphoramide mustard, the principal bifunctional DNA-crosslinking effector mediating antineoplastic and immunosuppressive activity, and electrophilic by-products that shape dose-limiting toxicity [1]. In contemporary clinical practice, CP remains embedded in multi-agent chemotherapy regimens and is retained as a high-efficacy induction option in severe immune-mediated diseases; notably, guideline-level recommendations continue to position CP as an established remission-induction agent in organ- or life-threatening ANCA-associated vasculitis and in proliferative lupus nephritis [2,3].

Alongside therapeutic benefit, CP has well-recognised urogenital toxicity, with urotoxicity representing a canonical clinical constraint. Hemorrhagic cystitis is mechanistically linked to urinary exposure of the urothelium to reactive metabolites (classically acrolein), which promotes oxidative stress, microvascular injury, and mucosal inflammation; accordingly, preventive practice incorporates “chemopreventive” strategies such as aggressive hydration/diuresis and thiol-based uroprotection (mesna), although regimen- and risk-dependent uncertainty persists regarding optimal prophylaxis and the magnitude of benefit in specific settings [4]. In parallel, nephrotoxicity is biologically plausible and widely operationalised in preclinical models as a tubulo-interstitial phenotype in which redox disequilibrium and secondary inflammatory/apoptotic amplification contribute to functional impairment; broader oncology-focused syntheses similarly emphasise that renal liability is a relevant adverse-effect axis for several cytotoxics, including alkylators, strengthening the rationale for mechanistically explicit nephroprotection strategies in vivo [5].

Mechanistically, CP nephrotoxicity is most commonly framed as a bioactivation-driven cascade in which reactive intermediates and downstream electrophiles impose thiol stress, amplify lipid peroxidation, and propagate secondary inflammatory and apoptotic signalling within the renal tubular compartment [6,7]. In this framework, oxidative imbalance is treated not simply as an epiphenomenon but as a proximate driver that can couple chemical injury to downstream biological amplification, including NF-κB-linked inflammatory activation and caspase-dependent cell death programmes [6,7,8,9]. Nevertheless, despite the biological plausibility of this cascade and the sheer volume of animal experiments, the field has not converged on a quantitatively defensible “core” phenotype or a robustly comparable evidence base, primarily because CP protocols vary widely in dose intensity and schedule, sampling windows range from hours to weeks, and endpoint selection and reporting formats are inconsistent across laboratories.

This fragmentation generates two persistent knowledge gaps that directly affect interpretation. First, the magnitude of functional injury is frequently discussed as though it were comparable across experiments, even though protocol variability can redefine the injury phenotype and its kinetics, making cross-study magnitudes non-portable without tight regimen matching. Second, the nephroprotection literature is particularly vulnerable to counterfactual ambiguity: in many designs, improvement is implicitly inferred from comparisons that do not isolate therapeutic reversal relative to CP-only comparators, thereby risking conflation of model induction dynamics with treatment effects. These conceptual gaps are compounded by a third, methodological barrier: incomplete numerical reporting. Preclinical meta-analysis typically requires tabulated group-level summary statistics (sample size, mean, and dispersion convertible to SD) at clearly defined time points, yet many CP nephrotoxicity studies report mechanistic outcomes only as figures or in non-harmonised semi-quantitative formats, reducing quantitative eligibility under conservative extraction rules and potentially skewing which endpoints become “meta-visible” [10,11,12,13].

The overarching objective was to integrate the disparate endpoint literature into a single mechanistic account anchored by the most reproducible meta-eligible signals and to translate that synthesis into pragmatic implications for preclinical study design and reporting. Prior syntheses of the CP nephrotoxicity literature, particularly those centred on antioxidant strategies, motivate a priori expectations that CP exposure produces consistent deterioration in classical functional markers accompanied by a robust renal oxidative signature, and that diverse interventions may converge on partial restoration of redox balance [6]. Accordingly, we hypothesised—in the restricted, synthesis-appropriate sense of pre-specified directional expectations—that CP would be associated with a reproducible oxidative shift in renal tissue alongside functional impairment, and that nephroprotective interventions, when evaluated against CP-only comparators and pooled without class ranking, would manifest as partial reversal across these core domains, while recognising that extreme between-experiment heterogeneity is intrinsic to CP protocol diversity and must be treated as a standardisation problem rather than as a basis for overconfident mechanistic ranking [13].

## 2. Materials and Methods

### 2.1. Review Design and Reporting Framework

This work was designed as a preclinical systematic review with planned quantitative synthesis (meta-analysis) of in vivo animal studies investigating cyclophosphamide (CP)-induced nephrotoxicity and its modulation by pharmacological or nutraceutical interventions. The methodological structure, data extraction, and reporting adhered to internationally recognised standards for animal systematic reviews, in compliance with the PRISMA 2020 statement and the SYRCLE risk-of-bias framework for animal studies, and took into account key elements of the ARRIVE 2.0 guidelines for reporting of animal research. The protocol—including the research question, eligibility criteria, search strategy, and planned analyses—was developed a priori before screening and data extraction and was archived in the Open Science Framework (OSF; https://doi.org/10.17605/OSF.IO/D9QUP) prior to manuscript submission. Any subsequent refinements (e.g., collapsing mechanistic markers into broader outcome domains when data were sparse) were defined before quantitative analyses and are explicitly documented in Section 3 and Supplementary Material.

### 2.2. Conceptual Framework and Analytic Contrasts (Model vs. Treatment Effects)

To prevent counterfactual ambiguity in nephroprotection claims and avoid conflating model induction dynamics with intervention effects, we analysed two contrasts separately. The model effect was defined as CP versus control (CP-only vs. untreated/vehicle controls), capturing the magnitude and direction of renal injury attributable to the CP regimen. The treatment effect was defined as intervention + CP versus CP-only, capturing incremental protection (or harm) attributable to the intervention relative to the appropriate CP comparator. Quantitative pooling was restricted a priori to outcomes with extractable tabulated group-level summary statistics (sample size, mean, and dispersion convertible to SD) at clearly defined post-CP time points; figure-only outcomes were retained for narrative synthesis but excluded from pooling under a prespecified no-digitisation constraint to preserve auditability and reduce extraction subjectivity. Functional outcomes were synthesised as mean differences after unit harmonisation (mg/dL), whereas oxidative endpoints were synthesised as standardised mean differences (Hedges’ g) to accommodate heterogeneous assay formats. Random-effects models were used throughout to account for expected between-study heterogeneity arising from CP regimen diversity, sampling windows, and endpoint measurement protocols.

### 2.3. Research Question and Eligibility Criteria

The research question was formulated using a PICO framework focused on preclinical models of CP-induced nephrotoxicity.

Population (P). Experimental animal models (rats, mice, rabbits, or other vertebrate species) exposed to CP in vivo, with explicit assessment of renal injury. Studies using non-vertebrate models, in vitro or ex vivo preparations (e.g., isolated kidney, cell lines), or exclusively human participants were excluded.

Intervention/Exposure (I). Administration of cyclophosphamide as the primary nephrotoxic insult, using any dose, route (e.g., intraperitoneal, oral), and schedule (single or repeated dosing), provided that the experimental design clearly allowed attribution of kidney injury to CP. Studies in which CP was only one component of a non-separable multidrug regimen without an identifiable CP-only arm were excluded.

Comparator (C). Healthy, untreated, or vehicle-treated control groups without CP exposure were required. For studies testing nephroprotective interventions, the minimal design had to include at least a normal/vehicle control group and a CP-only group; additional CP + intervention arms were considered as treatment groups.

Outcomes (O). To be eligible, studies had to report at least one quantitative indicator of kidney injury and/or mechanistic involvement in CP-induced nephrotoxicity. Outcomes were grouped a priori into the following:

- Functional renal markers: Blood urea nitrogen (BUN), serum creatinine, serum urea, uric acid, creatinine clearance, urine protein/albumin, urine output.
- Oxidative stress and antioxidant defence: Malondialdehyde (MDA) or other lipid peroxidation markers, reduced glutathione (GSH), oxidised glutathione (GSSG), total antioxidant capacity (TAC), superoxide dismutase (SOD), catalase (CAT), glutathione peroxidase (GPx), glutathione reductase, and related indices.
- Inflammatory mediators: TNF-α, IL-1β, IL-6, IL-10, NF-κB activation, COX-2 expression, myeloperoxidase (MPO) activity, NLRP3 inflammasome components, and other cytokines or chemokines.
- Apoptosis and fibrosis: Bax, Bcl-2, cleaved/total caspase-3 or caspase-9, TUNEL positivity, TGF-β1, collagen deposition, fibrotic scoring, and markers of extracellular matrix remodelling.
- Histopathological lesions: Semi-quantitative or descriptive scoring of glomerular, tubular, interstitial, and vascular changes (e.g., tubular necrosis, cast formation, interstitial oedema, inflammatory infiltrates, fibrosis).

Timeframe (T) and study type. Only full-length, peer-reviewed original research articles published between 1 January 2010 and 31 December 2025 in English or Russian were considered. Reviews, editorials, letters, conference abstracts without full data, and case reports were excluded. Studies focused primarily on non-renal organs (e.g., liver, heart, lung, testis, ovary, brain) without quantitative kidney outcomes were excluded, even if CP was used.

### 2.4. Information Sources and Search Strategy

A comprehensive literature search was conducted in the following databases: PubMed/MEDLINE, Scopus, Web of Science Core Collection, and Embase (Elsevier). Google Scholar was additionally used as a supplementary source to identify potentially relevant studies from regional or less-indexed journals.

The core search strategy combined controlled vocabulary and free-text terms for cyclophosphamide, nephrotoxicity, and key mechanistic domains (oxidative stress, inflammation, apoptosis, fibrosis), together with animal-model filters and exclusion terms for other target organs. An example PubMed query was (“cyclophosphamide-induced nephrotoxicity” OR “cyclophosphamide-induced kidney injury” OR “cyclophosphamide-induced renal injury” OR (“cyclophosphamide” AND (“nephrotoxicity” OR “renal toxicity” OR “kidney injury”))) AND (“oxidative stress” OR “inflammation” OR “apoptosis” OR “fibrosis”) AND (“rat” OR “rats” OR “mouse” OR “mice” OR “animal model” OR “in vivo”) NOT (“liver” OR “testis” OR “ovary” OR “lung” OR “heart” OR “brain”) (Supplementary Table S4).

Search filters restricted results to the years 2010–2025 and to articles in English or Russian. Equivalent Boolean logic was adapted to Scopus (TITLE-ABS-KEY syntax), Web of Science, and Embase (EMTREE terms plus free text and animal filters). Google Scholar searches used simplified combinations of the same core concepts and excluded non-renal targets using negative terms.

Across all sources, searches performed on 20 November 2025 (PubMed) and 25 November 2025 (Scopus and supplementary retrieval via Google Scholar/Elicit) yielded 57 records from PubMed, 59 from Scopus, and 32 additional records from Google Scholar/Elicit, for a total of 148 records prior to deduplication. All search results were imported into Zotero for reference management and deduplication. Because record-level exports in a uniform format were available for PubMed and Scopus, Supplementary Table S5 reports detailed cross-platform overlap and deduplication for the PubMed + Scopus subset; records obtained via other sources are reflected in the overall pre-/post-deduplication totals. The last searches were performed on 25 November 2025.

### 2.5. Study Selection

After automatic and manual deduplication in Zotero, 83 unique records remained for screening. Title and abstract screening were performed independently by two reviewers, who classified each record as “include,” “exclude,” or “uncertain” based on the predefined PICO and eligibility criteria. Full texts were retrieved for all records marked as “include” or “uncertain.”

At the full-text stage, the same reviewers independently assessed eligibility, documenting explicit exclusion reasons for ineligible studies (e.g., wrong organ, in vitro only, review article, no CP-only group, no renal outcomes). The main reasons for full-text exclusion were: (i) review or overview articles without primary in vivo data on cyclophosphamide-induced nephrotoxicity (including general antioxidant reviews, Panax ginseng overviews, and cyclophosphamide neurotoxicity-focused reviews); (ii) wrong population, organ, or exposure, such as studies centred on cisplatin-induced kidney injury, ovarian damage, cardiotoxicity, hepatotoxicity, or cyclophosphamide-induced urotoxicity/bladder toxicity without quantitative renal endpoints; (iii) imaging or mechanistic studies without an in vivo CP kidney injury model or without a nephroprotective intervention (e.g., EPR imaging of acute kidney injury, isolated urothelial/bladder preparations); and (iv) one glutamine study in which the test agent did not demonstrate nephroprotective effects on predefined functional and structural renal outcomes, despite partial modulation of oxidative stress markers.

**Figure 1:**
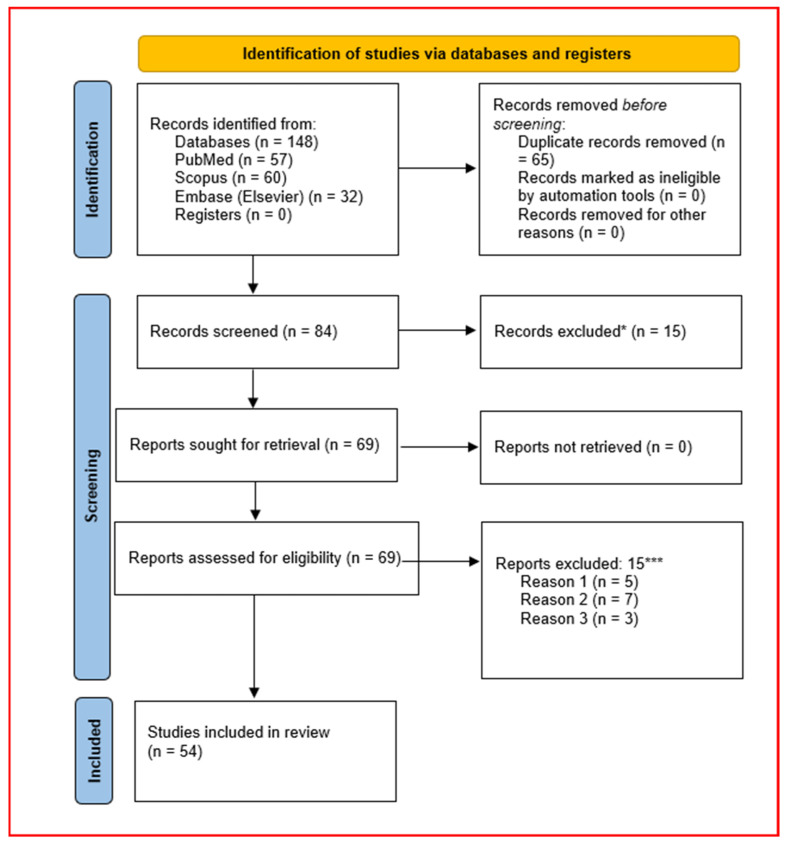
PRISMA 2020 flow diagram for new systematic reviews, which included searches of databases and registers only. Note: * refers to title/abstract screening exclusions. *** Reports excluded with reasons. Reason 1—Review/overview; no primary in vivo data on CP nephrotoxicity; Reason 2—Wrong population/organ/exposure; non-renal CP focus or different agent; Reason 3—Imaging/mechanistic studies without an in vivo CP kidney injury model and/or without a nephroprotective intervention.

In total, 15 full-text articles were excluded for these prespecified reasons. Ultimately, 54 studies fulfilled all eligibility criteria and were included in the qualitative synthesis; among these, only those providing complete numerical data (group means, dispersion, and sample sizes at clearly defined time points) were eligible for quantitative pooling in the meta-analysis, as described in Section 2.8.2 [8,9,14,15,16,17,18,19,20,21,22,23,24,25,26,27,28,29,30,31,32,33,34,35,36,37,38,39,40,41,42,43,44,45,46,47,48,49,50,51,52,53,54,55,56,57,58,59,60,61,62,63,64,65]. Disagreements at any screening stage were resolved by discussion; if consensus was not reached, a third senior reviewer adjudicated. The overall selection process, including the number of records identified, screened, excluded (with reasons), and included in the final synthesis, is summarised in a PRISMA 2020 flow diagram (Figure 1).

### 2.6. Data Extraction and Management

A standardised, piloted data extraction form was developed in Excel to ensure consistent capture of experimental and mechanistic details across studies. For each eligible article, two reviewers independently extracted the following information:

- Bibliographic details: First author, year of publication, journal, country or region.
- Study design and risk-of-bias-relevant features: Number of experimental groups; group size and total sample size; statement and method of randomization; blinding of investigators or outcome assessors; handling of exclusions and missing data; any sample size calculation.
- Animal model characteristics: Species, strain, sex, age category (e.g., juvenile, adult), baseline body weight, comorbid conditions (e.g., diabetes, hypertension), special diets or preconditioning protocols, and key housing conditions when reported (e.g., temperature, light–dark cycle, access to food and water). Sex was prespecified as a potential effect modifier for both CP nephrotoxicity (model effect) and intervention efficacy (treatment effect); where sex-stratified groups with extractable group-level statistics were reported, these strata were to be retained as separate comparisons and explored descriptively or via stratified analyses if sufficient in number.
- Cyclophosphamide protocol: CP dose(s) in mg/kg, route of administration (e.g., i.p., p.o.), frequency (single vs. repeated dosing), total duration of exposure, vehicle used for CP, and the time interval between CP administration and outcome assessment. Where described, confirmation that the model produced nephrotoxicity (e.g., elevation of BUN/creatinine, typical histological lesions) at the chosen dose/time point was recorded.
- Protective or test interventions: Identity and class of the nephroprotective agent (e.g., natural antioxidant, small-molecule inhibitor, clinically used drug), dose and dosing schedule, route and timing relative to CP (pre-treatment, co-treatment, or post-treatment), vehicle or control conditions for the intervention, and any mechanistic rationale articulated by the authors. CP-only experiments without any protective agent were coded as “CP only.”
- Functional renal outcomes: Serum BUN, creatinine, urea, uric acid; creatinine clearance; urine protein/albumin excretion; urine volume and electrolytes, where available. For each outcome and time point, we extracted group-level means, measures of dispersion (standard deviation or standard error), and sample sizes, along with *p*-values or other indicators of statistical significance.
- Biochemical outcomes: Quantitative markers of oxidative stress (MDA or equivalent lipid peroxidation products, reactive oxygen species indices), antioxidant defences (GSH, GSSG, GSH/GSSG ratio, SOD, CAT, GPx, total antioxidant capacity), and any other serum or tissue biochemistry relevant to nephrotoxicity. Direction (increase/decrease vs. control), magnitude of change, and whether the intervention significantly reversed CP effects were explicitly coded.
- Inflammatory, apoptotic, and fibrotic markers: Tissue or serum levels of TNF-α, IL-1β, IL-6, IL-10, MPO, NF-κB activation status, NLRP3 inflammasome components, Bax/Bcl-2 ratio, caspase-3 and caspase-9 expression/activity, TUNEL-positive cells, TGF-β1, collagen content, and any histological fibrosis scores.
- Histopathology: Type of staining (e.g., H&E, Masson’s trichrome, PAS), qualitative descriptions (e.g., tubular necrosis, cast formation, interstitial inflammation, glomerular congestion), and semi-quantitative scoring systems were used. We recorded whether the protective intervention prevented or reduced structural injury relative to CP-only animals and whether representative photomicrographs were provided.
- Molecular evidence and signalling pathways: mRNA and protein expression assessed by RT-PCR, Western blotting, immunohistochemistry or immunofluorescence; specific pathways probed (e.g., Nrf2/Keap1/HO-1, MAPK/ERK, NF-κB, JNK/p38, TGF-β/Smad); and the direction and significance of pathway modulation in CP vs. control, and intervention vs. CP groups.
- Data suitability for meta-analysis: For each study and outcome, we recorded whether complete numerical data (means, SD/SEM, *n* per group, clearly defined time point) were available to compute effect sizes. Outcomes lacking extractable numerical data (e.g., reported only graphically without scales or exact values) were flagged as “non-quantifiable” and used only in qualitative synthesis unless reliable digital extraction was feasible.
- Key conclusions and translational claims: Authors’ own interpretation regarding nephroprotection, proposed mechanisms, dose–response relationships, and any explicit statements on clinical relevance or limitations of the animal model.

The database search was executed by one reviewer using a prespecified strategy, and all retrieved records were exported and deduplicated before screening. Title/abstract screening and full-text eligibility assessment were performed independently by two reviewers. Disagreements at either stage were resolved by discussion; when agreement could not be reached, a third senior reviewer adjudicated. A Delphi process was not employed.

Data extracted independently were cross-checked and merged; discrepancies were resolved by consensus, with recourse to a third reviewer when necessary. If essential numerical data were missing or ambiguous, attempts were made to infer values from tables; when this was not possible, the outcome was excluded from quantitative pooling but retained in the narrative synthesis. Study-by-outcome accounting of tabulated statistics versus figure-only or missing reporting for the core endpoints is provided in Supplementary Tables S1–S3. Risk-of-bias judgements derived from the extracted methodological reporting items were compiled at the study level using SYRCLE and are presented as a domain-by-domain table in Supplementary Table S8, with a visual summary in Figure S1.

### 2.7. Assessment of Risk of Bias and Reporting Quality

Risk of bias was assessed at the study level using SYRCLE’s Risk of Bias tool for animal intervention studies, covering selection bias (sequence generation, baseline characteristics, allocation concealment), performance bias (random housing and blinding of caregivers/investigators), detection bias (random outcome assessment and blinding of outcome assessors), attrition bias (incomplete outcome data), reporting bias (selective outcome reporting), and other sources of bias. Each domain was judged as low, high, or unclear risk of bias based on the information reported in the Section 3 of the original publication. Because reporting was frequently incomplete, ‘unclear’ was used when the methodological safeguard was not explicitly described. A complete domain-by-domain judgement for all included studies is provided in Supplementary Table S8, accompanied by a visual summary of domain-level judgements (Figure S1).

### 2.8. Data Synthesis and Statistical Analysis

#### 2.8.1. Qualitative Synthesis

All included studies were first summarised descriptively. We constructed overview tables detailing animal species and strains, CP dosing regimens, co-interventions, main mechanistic domains examined (oxidative stress, inflammation, apoptosis, fibrosis), and principal functional and histopathological outcomes.

Narrative synthesis was structured around:

- CP nephrotoxicity models (species, dose levels, exposure duration, reproducibility of injury).
- Oxidative stress and antioxidant defence imbalance.
- Inflammatory and innate immune activation (NF-κB- and NLRP3-related pathways).
- Apoptotic and fibrotic remodelling of renal tissue.
- Classes of nephroprotective interventions (natural products, clinically used drugs, enzyme modulators, signalling-pathway-targeted compounds) and their mechanistic profiles.

Where appropriate, patterns across different compounds acting on similar pathways (e.g., Nrf2 activators, NF-κB inhibitors) were explicitly compared.

#### 2.8.2. Quantitative Synthesis (Meta-Analysis)

A quantitative synthesis was undertaken for pre-specified core outcomes that are both frequently reported and sufficiently comparable across in vivo rodent models of cyclophosphamide (CP) nephrotoxicity: serum creatinine and serum urea/BUN as functional indices, and renal malondialdehyde (MDA/TBARS) and reduced glutathione (GSH) as principal oxidative injury and antioxidant defence markers. Meta-analysis was restricted to experiments for which group-level summary statistics (sample size, mean, and dispersion convertible to SD) were extractable from the full text at a clearly defined post-CP time point. To preserve reproducibility, outcomes reported exclusively in figures without numerical tables were retained for qualitative synthesis but were not quantitatively pooled (i.e., no figure digitisation was used for the primary meta-analysis) (Tables S1–S3). Evidence base size per endpoint and contrast (k comparisons, contributing studies, and unique post-CP time points) is summarised in Supplementary Table S1. Study-by-outcome accounting of tabulated versus figure-only/missing reporting that determined quantitative admissibility under the no-digitisation rule is provided in Supplementary Table S2. Small-study effects were evaluated using Egger’s regression only where k ≥ 10 (Supplementary Table S3).

Two contrasts were analysed separately to avoid conflation of model induction with nephroprotective efficacy: the “model effect” (CP vs. untreated/vehicle controls) and the “treatment effect” (intervention + CP vs. CP-only). For functional endpoints, values were harmonised to common units prior to pooling and analysed as mean differences (MD). Serum creatinine reported in µmol/L or mmol/L was converted to mg/dL (1 mg/dL = 88.42 µmol/L; mmol/L converted via multiplication by 1000/88.42). Serum urea reported in mmol/L was converted to mg/dL (1 mmol/L = 6.006 mg/dL). BUN and urea were treated as distinct outcomes unless a defensible conversion was available; where harmonisation was not justifiable, pooling was either performed separately or not performed. For oxidative endpoints, unit harmonisation was not considered defensible across assay formats and reporting bases; therefore, effects were pooled as standardised mean differences using Hedges’ g with small-sample correction.

Random-effects models were used throughout as the default analytic framework given anticipated between-study heterogeneity in CP regimen intensity, sampling time, animal characteristics, and intervention dosing. Between-study variance (τ^2^) was estimated using the DerSimonian–Laird method, and pooled effects are reported with 95% confidence intervals; heterogeneity was quantified using I^2^ and τ^2^. When multiple intervention arms shared a single CP-only comparator group, unit-of-analysis inflation was avoided by splitting the comparator sample size across arms for variance computation while retaining the original group means and SDs. When studies reported multiple post-CP time points, extraction prioritised the terminal (protocol-end) assessment when clearly designated; otherwise, the extracted time point recorded for the experiment was used.

Prespecified subgroup analyses were conducted only where replication was sufficient to support inference (minimum of two comparisons from at least two independent studies per subgroup level). Moderator analyses focused on CP regimen type (acute single-dose vs. repeated/subacute or other regimens) and broad post-CP sampling windows (≤24 h, 2–7 days, >7 days), with “not reported” time points retained only for descriptive accounting and excluded from inferential subgroup testing. Small-study effects were explored using Egger’s regression test only when at least ten comparisons were available for a given outcome–contrast combination, recognising limited power and potential distortion under substantial heterogeneity. All analyses were performed in Python 3.11.4 using standard scientific libraries for numerical computation and plotting; forest plots were generated for pools with sufficient eligible comparisons, and a complete study-level effects table was exported to ensure the reproducibility and auditability of the pooled estimates. Evidence-based size per endpoint and per contrast (CP vs. control; intervention + CP vs. CP) is summarised in Supplementary Table S1.

#### 2.8.3. Subgroup and Sensitivity Analyses

Where data permitted, we planned subgroup analyses exploring the following:

- Species (rat vs. mouse).
- CP dosing paradigm (single high-dose vs. repeated lower doses).
- Timing of intervention (pre-treatment vs. co-/post-treatment).
- Mechanistic class of intervention (e.g., primarily antioxidant vs. primarily anti-inflammatory vs. mixed).
- Risk-of-bias strata (studies with predominantly low vs. high/unclear risk of bias in key domains).

Sensitivity analyses were envisaged to examine the robustness of pooled effect estimates:

- Excluding studies at high risk of bias in randomization or outcome assessment.
- Excluding clear statistical outliers.
- Using fixed-effect instead of random-effects models.

Potential small-study or publication bias was to be explored for meta-analyses including at least ten studies, using visual inspection of funnel plots and, where appropriate, Egger’s regression test.

### 2.9. Role of Funding and Conflicts of Interest

During data extraction, any information on study funding sources and conflict-of-interest statements reported by the original authors was recorded, with particular attention to sponsorship from manufacturers of tested interventions. Although not used as a formal risk-of-bias domain, this information contributed to the contextual interpretation of results and is summarised descriptively in Section 3.

## 3. Results

### 3.1. Methodological Reporting

Methodological reporting was heterogeneous across the included evidence base. A statement indicating random allocation to groups was present in 35/54 studies (64.8%), while any form of blinding (investigators and/or outcome assessors) was reported in 13/54 studies (24.1%). No study reported a formal sample size calculation or power analysis (0/54, 0.0% for both). Study-level SYRCLE risk-of-bias judgements are summarised in Supplementary Table S8 and Figure S1.

### 3.2. Experimental Models and Nephroprotective Interventions

Most studies used adult male Wistar or Sprague–Dawley rats, with Swiss albino, Kunming, and BALB/c mice as the main murine strains; only a small subset did not specify strain/stock or reported mixed stocks [15,22,30,33,45,57,64] (Table 1 and Table S6). Comorbidity models (e.g., diabetes or hypertension) were not a defining feature of the included evidence base; where explicitly reported, animals were young adults without induced comorbidities and were maintained under conventional laboratory housing (controlled temperature/humidity, 12:12 h light–dark cycle, standard chow and water ad libitum). Reporting of these husbandry parameters, however, was inconsistent across studies. Experiments were overwhelmingly performed in males, while explicit inclusion of females or mixed-sex cohorts was uncommon, and sex-specific analyses were rarely reported. Consequently, sex-stratified quantitative synthesis was not feasible because sex-stratified groups and extractable sex-specific outcome statistics were generally unavailable; sex was therefore handled descriptively as a study characteristic and interpreted as a limitation. Table 1 summarises the design and outcome coverage of all included studies (*n* = 54).

CP was consistently used as the index nephrotoxicant, but dosing regimens varied in intensity and scheduling. Most studies employed an acute high-dose paradigm, typically a single intraperitoneal bolus of 150–200 mg/kg, with outcomes assessed within the early post-exposure window (approximately 24–72 h) to capture rapid-onset renal injury [39,47,49,56,60,63]. A smaller subset used short-course repeated dosing, including repeated intraperitoneal administration over several days in rats and, in murine models, daily i.p. dosing for 3–5 days [18,35,53,58,63]. Some designs retained an acute high-dose CP bolus but incorporated extended prophylactic intervention windows and/or dual-organ endpoint panels, including concurrent assessment of bladder injury consistent with haemorrhagic cystitis [52], while other less common schedules applied lower-dose multi-day exposure and expanded urinary functional profiling [40,43,64]. Across regimens, nephrotoxicity was confirmed by convergent evidence from functional markers (serum creatinine and urea/BUN, and occasionally creatinine clearance or proteinuria), oxidative and inflammatory indices, and histopathological documentation of tubular-predominant injury with variable glomerular involvement [31,47,53,59].

Geographically, the evidence base showed pronounced clustering, with a substantial proportion of studies originating from laboratories in the Middle East and North Africa—particularly Iran, Saudi Arabia, Turkey, and Egypt—and additional contributions from South and East Asia (notably India and China), e.g., [16,22,32,40,47,53,62]. Despite this geographic concentration, the experimental logic and outcome domains were broadly similar across studies, with a strong emphasis on classical biochemical markers and histopathological evaluation.

Nephroprotective interventions spanned several mechanistic classes. A first cluster comprised conventional antioxidants, micronutrients, and related metabolic/nutritional interventions, including vitamin E, selenium/seleno-L-methionine, glutathione (including nano-carrier formulations), whey protein isolate, and aminoguanidine [34,39,43,50,51,53,60,63]. A second, numerically dominant cluster evaluated plant-derived small molecules, predominantly flavonoids and polyphenols, alongside related phytochemicals, including naringin, naringenin, resveratrol, chrysin, quercetin, formononetin, berberine, ellagic acid, bergapten, herbacetin, irigenin, pterostilbene, nerolidol, and betulinic acid [8,9,22,32,33,36,37,40,54,56,57,58,61]. A further subset of studies investigated crude plant extracts and complex formulations, including *Ocimum gratissimum*, *Capparis spinosa*, *Murraya koenigii*, *Elaeagnus angustifolia* fruit extract, propolis, *Picrorhiza kurroa* iridoid fractions, and the Chinese formulation Huaiqihuang [16,19,28,35,44,47,59].

Repurposed small-molecule drugs and approved clinical agents formed a third intervention category, including metformin, alogliptin, zofenopril (alone or combined with thymoquinone), nebivolol, tolvaptan, and tranilast, typically selected for their established metabolic, anti-inflammatory, or antifibrotic profiles [20,25,26,27,29,45]. A smaller subset evaluated nanoparticles and inorganic agents (e.g., cerium oxide nanoparticles and boric acid), generally framed around redox modulation and anti-apoptotic signalling [23,63]. Several reports investigated peptides, protein-based preparations, and other non-classical interventions, including low-molecular-weight marine peptides, plasma proteins from marine organisms, lactoferrin, and exogenous hydrogen sulfide donors, frequently contextualised by the modulation of Nrf2/HO-1, NF-κB, and inflammasome-related pathways [14,17,21,31].

For quantitative synthesis, stratification was prespecified by species (rats vs. mice) and CP regimen (acute single high-dose bolus vs. short-course repeated dosing; atypical schedules treated separately) and, where data density permitted, by intervention class. In practice, subgroup (moderator) analyses could be implemented only for a limited subset of core outcomes because replication within strata was often insufficient, leaving residual heterogeneity unresolved.

Taken together, these protocols produced a broadly consistent pattern of CP-induced kidney injury across four outcome domains: functional markers, oxidative–antioxidant balance, inflammatory and apoptotic mediators, and histopathological (including, where assessed, fibrotic) changes.

### 3.3. Outcome Domains and Mechanistic Readouts

Before detailing individual outcome domains, it is important to emphasise that the read-outs summarised in the subsections below are not independent “markers” in a strict mechanistic sense, but time- and assay-dependent snapshots of a continuous injury cascade. In CP nephrotoxicity, reactive metabolites are typically associated with an early perturbation of tubular and microvascular redox balance, followed by activation of inflammatory signalling (including NF-κB-linked pathways), leukocyte recruitment, and apoptosis; with sufficient duration or cumulative exposure, these processes may progress towards extracellular matrix remodelling, fibroblast activation, and fibrosis. The subdivision into functional, oxidative–antioxidant, inflammatory/apoptotic, fibrotic, and novel biomarker domains therefore serves primarily as an organisational and quantitative framework. Conceptually, these endpoints represent successive and partially overlapping layers of the same CP-induced injury process, with their relative prominence determined by sampling windows and analytical panels rather than sharply separated pathogenetic modules.

Across the 54 included studies, CP-induced renal injury was characterised using recurring outcome domains: (i) functional kidney markers, primarily serum creatinine and serum urea or BUN, with occasional urinary indices; (ii) classical oxidative–antioxidant biochemistry; (iii) inflammatory and apoptotic mediators; (iv) histopathological changes and, where assessed, fibrosis-oriented read-outs, sometimes accompanied by targeted signalling analyses; and (v) novel kidney injury biomarkers such as cystatin C, kidney injury molecule-1 (KIM-1), and neutrophil gelatinase-associated lipocalin (NGAL). Core functional endpoints were available in a subset of studies (serum creatinine and serum urea each reported in 15 studies), while oxidative core endpoints (kidney MDA and kidney GSH) were available in 13 studies, with a substantial fraction providing figure-only or otherwise non-extractable statistics under the no-digitisation rule (Supplementary Table S2). Beyond these core outcomes, reporting of inflammatory, apoptotic, fibrotic, signalling, and novel biomarker panels was heterogeneous and typically limited to a small number of preselected markers.

#### 3.3.1. Functional Kidney Outcomes

Functional assessment, where available, relied predominantly on serum creatinine and serum urea or BUN. Studies providing extractable tabulated statistics for serum creatinine and serum urea were available in 15 studies each (Supplementary Table S2), and these markers generally increased following CP exposure, supporting clinically interpretable nephrotoxicity in the subset reporting functional endpoints (e.g., [22,47,53,61]) (Table 2). However, creatinine responses were not uniform across designs: several protocols documented clear biochemical and histological injury with only modest or non-significant creatinine changes, highlighting the limited sensitivity of traditional filtration markers in early post-CP windows and motivating the use of tubular injury biomarkers as adjuncts [20,32,43,60].

The functional evidence base encompassed both acute single-bolus models (commonly 150–200 mg/kg i.p., early follow-up) and short-course repeated dosing regimens over several days (Table 1). To distinguish coverage from extractability, Table 2 summarises the reporting frequency of creatinine and urea/BUN across CP regimen categories and timepoints (including studies with figure-only reporting), whereas Supplementary Table S2 summarises the availability of tabulated statistics suitable for quantitative synthesis. In acute protocols, pronounced oxidative and structural injury frequently developed within 24–48 h, but the magnitude of creatinine and urea/BUN elevation varied, with some studies reporting robust increases in both markers (e.g., [17,47,56]) and others showing limited creatinine responsiveness despite clear injury signals [20,32,60]. Where longer or repeated dosing schedules were used, functional impairment tended to be more consistent and, when assessed, was accompanied by reduced creatinine clearance, proteinuria/albuminuria, or altered urinary electrolyte handling, indicating a more established decline in renal function [35,45,53,61] (Table 2).

Across mechanistic classes, nephroprotective interventions generally produced functional benefit in studies reporting serum creatinine and/or urea/BUN. Classical antioxidants and micronutrients (vitamin E, selenium/seleno-L-methionine, aminoguanidine, glutathione formulations, and whey protein isolate) typically attenuated CP-associated elevations in creatinine and urea/BUN, shifting values towards control levels [34,39,51,53,60]. A broad range of flavonoids and polyphenols—including naringin, naringenin, hesperidin, quercetin, resveratrol, berberine, ellagic acid, nerolidol, bergapten, and irigenin—showed similar functional protection, with co-treated groups usually exhibiting lower creatinine and/or urea/BUN than their CP-only counterparts and, in some regimens, approaching near-normalisation [8,22,33,37,40,46,54,56]. Whole-plant extracts and traditional formulations—such as *Ocimum gratissimum*, *Capparis spinosa*, *Murraya koenigii*, *Elaeagnus angustifolia*, propolis, and Huaiqihuang—followed the same overall pattern, mitigating CP-associated functional impairment and, where assessed, improving urine output and proteinuria indices [16,19,35,44,47,59]. Repurposed clinical agents (metformin, alogliptin, zofenopril ± thymoquinone, tranilast, and tolvaptan) likewise improved functional indices, attenuating CP-induced elevations in creatinine/urea/BUN and normalising, when measured, urinary clearance and electrolyte handling [20,25,26,29,45]. Nanoparticles and inorganic agents (e.g., cerium oxide nanoparticles, boric acid) and hydrogen sulfide donors also exhibited functional protection in the studies reporting these endpoints [23,31,63].

Beyond conventional filtration markers, several studies incorporated more sensitive functional readouts. Dobrek et al. quantified urine volume, pH, sodium and potassium excretion, and urinary urea/uric acid alongside urinary NGAL-1, demonstrating an acute kidney injury-compatible pattern and highlighting NGAL-1 as an early functional/tubular injury indicator [64]. Other studies included cystatin C, KIM-1, and composite tubular injury indices; notably, at least one experiment reported increases in tubular biomarkers despite creatinine and urea remaining within normal ranges, underscoring both the limited sensitivity of traditional filtration markers and the added value of emerging tubular injury indicators [20,26,34]. Collectively, these data support serum creatinine as a primary functional endpoint, with urea/BUN, creatinine clearance, proteinuria/albuminuria, and tubular biomarkers (NGAL, KIM-1, cystatin C) as key secondary outcomes for quantitative synthesis in the present review and for improved functional phenotyping in future preclinical studies.

#### 3.3.2. Oxidative Stress and Antioxidant Defences

Oxidative stress was the most consistently interrogated proximal mechanistic domain in the CP-induced injury cascade. Across the included evidence base, the vast majority of studies reported at least one oxidative or antioxidant parameter (Table 1, Table 3 and Table S6). The most frequently used core read-outs were renal lipid peroxidation indices—typically malondialdehyde (MDA) or thiobarbituric acid reactive substances (TBARS)—paired with at least one endogenous antioxidant measure, most commonly reduced glutathione (GSH) and/or activities of superoxide dismutase (SOD), catalase (CAT) and glutathione peroxidase (GPx) (Table 3). In a substantial subset, these markers were complemented by nitrosative stress indices (nitric oxide or nitrite/nitrate, NO/NOx), global redox measures such as total oxidant status (TOS), oxidative stress index (OSI) and total antioxidant capacity/total antioxidant status (TAC/TAS or T-AOC), and less frequently by additional endpoints including hydrogen peroxide and oxidative DNA damage markers (e.g., 8-OHdG) [20,35,39,40,42,49,53,60,63].

**Figure 2:**
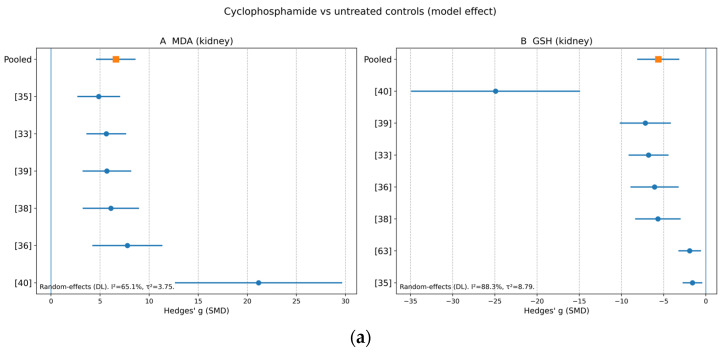

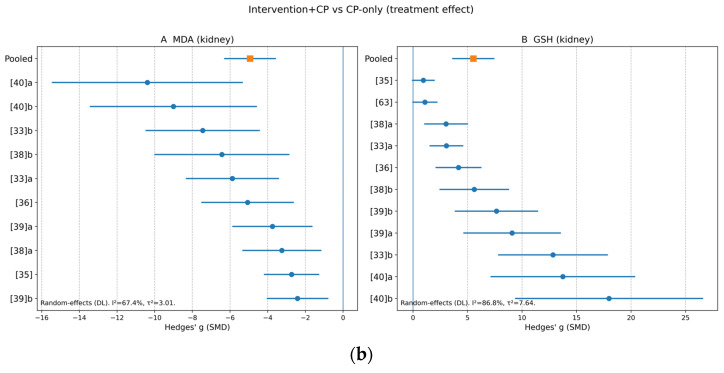
(**a**) Oxidative stress endpoints in renal tissue: model effect (cyclophosphamide vs. untreated controls). Panel A shows the pooled effect of cyclophosphamide exposure versus untreated controls on renal malondialdehyde (MDA) (CP vs. Control). Panel B presents the corresponding contrast for renal reduced glutathione (GSH). Effect sizes are expressed as Hedges’ g (standardised mean difference, SMD) with 95% confidence intervals and were estimated using random-effects models. Positive SMD values indicate higher levels in the CP group relative to controls, whereas negative SMD values indicate lower levels. Accordingly, CP exposure is expected to increase MDA and decrease GSH. Individual points represent study-level estimates (some studies contribute more than one estimate when multiple eligible arms are reported), and the pooled estimate is shown as a summary marker. (**b**) Oxidative stress endpoints in renal tissue: treatment effect (intervention + CP vs. CP-only). Panel A shows the pooled effect of nephroprotective interventions administered with cyclophosphamide versus cyclophosphamide alone on renal malondialdehyde (MDA) (Intervention + CP vs. CP). Panel B presents the corresponding contrast for renal reduced glutathione (GSH). Effect sizes are expressed as Hedges’ g (standardised mean difference, SMD) with 95% confidence intervals and were estimated using random-effects models. Negative SMD values indicate lower MDA levels in the intervention + CP group relative to CP-only, whereas positive SMD values indicate higher GSH levels, consistent with attenuation of oxidative injury and restoration of antioxidant capacity. Individual points represent study-level estimates, and the pooled estimate is shown as a summary marker. “Studies shown in the forest plots: [33,35,36,38,39,40,63].

Across species and strains, and irrespective of whether CP was administered as a single high dose or under repeated dosing schedules, the direction of change was broadly consistent. Where measured, CP increased MDA/TBARS and other oxidant indices (including NO/NOx, TOS and OSI) and decreased GSH and antioxidant enzyme activities (SOD, CAT, GPx and related defences), supporting a shared proximal mechanism of redox disequilibrium and lipid peroxidation-linked tubular injury [18,23,31,49,51,53,60,62,63] (Figure 2a,b).

Nephroprotective interventions from diverse mechanistic classes generally attenuated this oxidative signature in studies reporting these endpoints. Classical antioxidants and redox-modulating agents—including vitamin E, selenium/seleno-L-methionine, aminoguanidine, melatonin, exogenous hydrogen sulphide donors and glutathione delivered as nanostructured lipid carriers—tended to restore GSH and antioxidant enzyme activities and to lower MDA/TBARS and composite oxidant indices where quantified [31,34,39,47,49,51,53,60]. A broad spectrum of flavonoids and polyphenols (including naringin/naringenin, hesperidin, quercetin, resveratrol, berberine, ellagic acid, herbacetin, formononetin, bergapten, irigenin, sinapic acid and related phenylpropanoids/coumarins) showed similar antioxidant profiles, with co-treated groups generally exhibiting lower MDA/TBARS and higher GSH and antioxidant enzyme activities than CP-only cohorts and, when assessed, lower TOS/OSI and higher TAC/T-AOC [8,9,22,24,33,36,37,40,54,56,57,61]. Whole-plant extracts and traditional formulations—including *Ocimum gratissimum*, *Capparis spinosa*, *Murraya koenigii*, *Elaeagnus angustifolia*, *Lavandula officinalis*, ginger extract, Huaiqihuang, olive leaf extract and marine peptide preparations—largely reproduced this pattern, reducing peroxidation markers and strengthening endogenous antioxidant systems; in studies co-reporting renal function, these biochemical shifts often paralleled improvements in creatinine/urea and histological injury scores [14,15,16,17,19,35,42,48,59,62]. Repurposed clinical agents (metformin, alogliptin, zofenopril ± thymoquinone, tolvaptan and tranilast) similarly mitigated oxidative read-outs and restored antioxidant defences in the studies assessing these outcomes, frequently alongside improvements in conventional renal function and/or tubular injury biomarkers [20,25,26,29,45]. Nanoparticles and inorganic agents—particularly cerium oxide nanoparticles and boric acid—also showed redox-modulating activity, lowering MDA and NO/NOx and increasing GSH and antioxidant enzyme activities alongside reduced apoptotic signalling and amelioration of histological damage [23,63]. Notably, some short-term high-dose models demonstrated substantial oxidative and structural injury in the relative absence of marked changes in serum creatinine or urea, underscoring that oxidative endpoints may be more sensitive to early CP-induced damage than conventional filtration markers [43,49].

Mechanistic work converged on several redox-sensitive signalling axes. In targeted studies, multiple interventions—pyrroloquinoline quinone, marine and crustacean-derived peptides, Huaiqihuang, olive leaf extract, glutathione nanocarriers and lactoferrin—were reported to activate Nrf2 and downstream targets (HO-1, NQO1, GCLM) while attenuating NF-κB and, in some cases, NLRP3 inflammasome components [14,18,21,31,42,59,62]. Collectively, these findings support a recurring cytoprotective programme whereby structurally diverse nephroprotective strategies shift the balance from oxidative and nitrosative stress towards enhanced antioxidant capacity with coupled attenuation of downstream inflammatory signalling. As assay platforms and reporting units varied across studies, MDA/TBARS and GSH remain the most practical primary oxidative endpoints for quantitative synthesis (as standardised mean differences), while SOD, CAT, GPx, TAC/T-AOC and composite indices (TOS, OSI) provide robust secondary markers of CP-related oxidative injury and its attenuation by candidate interventions.

#### 3.3.3. Inflammatory and Apoptotic Mediators

Consistent with the redox perturbations described in Section 3.3.2, inflammatory read-outs were reported in a substantial subset of the evidence base. In the current extraction, at least one explicitly coded inflammatory mediator or pathway marker (e.g., cytokines, NF-κB-linked indices, MPO, inflammasome- or MAPK-associated signalling) was reported in a sizeable fraction of included studies, and pro-inflammatory cytokines (most commonly TNF-α, IL-1β and/or IL-6) were frequently co-measured alongside oxidative stress indices. This co-reporting supports a coherent injury sequence in which CP-associated oxidative imbalance is coupled to secondary inflammatory activation, rather than representing an independent downstream module [8,23,31,32,58,59]. Where quantified, CP exposure was generally associated with increased renal and/or systemic TNF-α, IL-1β and IL-6, and in targeted mechanistic studies, this cytokine induction co-occurred with evidence of NF-κB pathway activation (assessed variably as p65/IKK phosphorylation, nuclear translocation, or altered mRNA/protein abundance) and increased MPO activity as a proxy for leukocyte recruitment.

Across intervention classes, nephroprotective strategies largely attenuated inflammatory read-outs when these were measured. Multiple polyphenols and flavonoids (e.g., nerolidol, betulinic acid, formononetin, bergapten, protocatechuic acid), plant-derived mixtures (including ginger-derived preparations and *Picrorhiza kurroa* fractions), and peptide-based preparations were reported to reduce TNF-α, IL-1β and IL-6 and to dampen inflammatory signalling indices; in subsets, partial restoration of anti-inflammatory mediators such as IL-10 was also described [8,15,22,32,36,42]. Repurposed pharmacological agents and biologicals—including alogliptin, tranilast, metformin, zofenopril-based regimens, lactoferrin and Huaiqihuang—showed overlapping anti-inflammatory profiles in targeted experiments, frequently reported as coordinated down-regulation of NF-κB-linked signalling and, in smaller subsets, modulation of inflammasome components and/or upstream MAPK cascades [20,21,25,26,29,59]. Importantly, in most studies that assessed both domains, improvement in cytokine and pathway indices occurred in parallel with attenuation of MDA/TBARS, restoration of GSH, and partial recovery of antioxidant enzyme activities, reinforcing the mechanistic coupling between oxidative and inflammatory endpoints.

Apoptotic markers were also reported in a substantial subset of the mechanistic dataset, most commonly as caspase-3 activity or expression, Bax/Bcl-2 ratios, cytochrome c–related indices, TUNEL staining, or composite apoptotic scores. Across models, CP exposure typically increased caspase-3 (and in some experiments caspase-9 and/or caspase-8), shifted Bax/Bcl-2 towards a pro-apoptotic balance, and increased TUNEL-positive tubular cell counts, supporting activation of tubular cell death pathways compatible with a predominantly mitochondria-linked execution programme [9,32,56,57,58]. Interventions spanning diverse classes—including herbacetin and related flavonoids, iridoid fractions, sinapic acid-type phenolics, cerium oxide nanoparticles, metformin, bergapten, quercetin, lactoferrin and Huaiqihuang—were generally reported to reduce caspase-3 activation, decrease Bax and/or increase Bcl-2 expression, and lower TUNEL positivity, aligning improvements in oxidative and inflammatory read-outs with reduced tubular cell death [9,22,23,25,28,29,57,59].

Methodologically, the inflammatory and apoptotic evidence is more heterogeneous than the functional and core oxidative datasets. Marker panels, assay platforms, normalisation strategies and sampling time points vary widely across studies, and frequently reported endpoints are often presented in non-comparable units or semi-quantitative formats. Consequently, unlike serum creatinine/urea and the recurrent oxidative indices (MDA/TBARS and GSH), no single inflammatory or apoptotic endpoint is reported with sufficient frequency and unit comparability to support a robust pooled quantitative synthesis across the full evidence base. These domains are therefore synthesised primarily qualitatively (or semi-quantitatively where appropriate), interpreted in conjunction with the more standardised functional and oxidative outcomes, and used to map recurring mechanistic linkages rather than to generate pooled effect sizes. The dominant patterns are summarised in Table 4.

#### 3.3.4. Fibrosis, Signalling Pathways, and Histopathology

Fibrotic remodelling and other late structural sequelae were reported less consistently than functional, oxidative, or inflammatory outcomes. Across the included evidence base, fibrosis-oriented read-outs were documented in a minority of studies and were often expressed as qualitative histopathological descriptors rather than standardised quantitative measures. Within the extracted fields, profibrotic mediators such as TGF-β and collagen/ECM deposition indices were only sporadically reported, and dedicated fibrosis markers (e.g., α-SMA, hydroxyproline) were not consistently captured as recurrent outcomes in the current extraction framework; these endpoints, therefore, cannot be treated as common fibrosis read-outs without full-text verification and explicit recoding (Table 5).

Across the subset of intervention experiments that did evaluate fibrosis-related indices, diverse nephroprotective agents—including selected small molecules and polyphenols, plant-derived preparations, and peptide-based interventions—were reported to attenuate matrix-associated pathology and/or profibrotic signalling, typically in parallel with improvements in oxidative stress indices and histological injury severity [8,14,18,22,26,28,29,30,42]. Mechanistic coverage, however, was heterogeneous and is best represented as “reported in targeted studies” rather than as a uniform pathway panel. Within the extracted evidence, TGF-β/SMAD signalling was explicitly linked to fibrosis-related outcomes in only isolated reports, while ERK1/2, Wnt/β-catenin, p38 MAPK and PI3K/Akt were each assessed in limited, study-specific contexts. In contrast, NF-κB indices were frequently recorded across the dataset, but this predominantly reflects their use as an inflammatory stress axis rather than a fibrosis-specific endpoint and should be interpreted accordingly unless explicitly connected to matrix remodelling outcomes.

Histopathology was consistently reported across the evidence base. Routine H&E evaluation was explicitly described in most studies, with PAS and Masson’s trichrome used in smaller subsets, and immunohistochemistry variably referenced. Since “IHC/IF” is occasionally reported generically, it should be retained as a mapped domain only when supported by explicit Methods descriptions rather than inferred from general statements. The lesion spectrum aligns with a typical CP injury profile: tubular epithelial degeneration and necrosis with vacuolisation, tubular dilatation and cast formation, prominent interstitial inflammatory infiltration/congestion, and variable glomerular alterations [27,53,58,59,60]. Across intervention classes, agents generally reduced lesion severity and improved semi-quantitative injury read-outs where these were reported, providing structural corroboration for functional and molecular improvements; however, language implying “near-normal architecture” should be reserved for studies demonstrating statistically supported normalisation rather than descriptive improvement.

Overall, the outcome architecture remains internally coherent across the dataset: CP produces reproducible structural pathology together with convergent oxidative, inflammatory, apoptotic, and (less consistently assessed) fibrotic features, and candidate interventions tend to ameliorate these abnormalities (Table 5). From a quantitative synthesis perspective, the frequency and relative standardisation of creatinine/urea (BUN) and core oxidative indices (MDA/TBARS, GSH) support their prioritisation for meta-analysis, whereas signalling read-outs and histology-based endpoints will more reliably inform mechanistic narrative synthesis unless harmonised scoring systems and variance metrics are available [27,30,33,39,60].

#### 3.3.5. Novel Kidney Injury Biomarkers

A limited but methodologically important subset of the included literature moved beyond serum creatinine and urea/BUN and quantified emerging kidney injury biomarkers—most commonly cystatin C, kidney injury molecule-1 (KIM-1), and neutrophil gelatinase-associated lipocalin (NGAL)—in CP nephrotoxicity models [8,9,15,20,22,26,32,35,46,49,54,61,64]. These endpoints remain underutilised relative to classical renal function tests, and their reporting is heterogeneous in terms of biological matrix (serum/plasma vs. urine vs. renal tissue/expression), assay format (concentration-based measurement vs. expression/localisation read-outs), sampling time windows, and reporting units (Table 6). As a result, biomarker-specific conclusions are best interpreted as mechanistically informative signals within a minority of studies rather than as fully generalisable quantitative endpoints across the evidence base.

Despite limited coverage, the direction of effects was broadly consistent across studies that measured these biomarkers. CP exposure increased cystatin C, where it was measured, and robustly elevated the tubular injury markers KIM-1 and NGAL, consistent with proximal tubular stress/injury and tubulointerstitial damage [9,20,26,32,46,49,54]. Importantly, several experiments used these markers to resolve injury dynamics that are only partially captured by creatinine/urea alone, either by demonstrating larger relative biomarker shifts under comparable CP exposure or by detecting injury signals in settings where conventional filtration markers were modest, inconsistent, or statistically non-significant [20,26,32]. This pattern is biologically plausible: cystatin C reflects filtration and tubular handling, whereas KIM-1 and NGAL more directly index tubular epithelial injury and stress responses.

Intervention effects on novel biomarkers were broadly concordant with—and in some designs appeared more sensitive than—changes in creatinine/BUN. For example, alogliptin attenuated CP-induced elevations of cystatin C, KIM-1, and NGAL alongside improvements in oxidative stress indices, inflammatory read-outs, and histopathology [26]. Comparable normalising trends for one or more biomarkers were reported for polyphenolic and plant-derived interventions (e.g., *Ocimum gratissimum* extract, berberine, herbacetin-type flavonoids, protocatechuic acid) and for mechanism-oriented regimens that also modulated inflammatory/apoptotic signalling [8,9,15,22,32,35,54]. Where histology was co-reported, biomarker attenuation generally co-occurred with reduced tubular injury and inflammatory infiltration, supporting interpretability as integrated injury/response indicators rather than isolated laboratory signals [9,22,26,46].

From a synthesis standpoint, these biomarkers improve temporal and mechanistic resolution and have clear translational relevance; however, the current evidence base is not configured for robust pooled quantitative analysis. Many studies do not specify analytic platforms, matrices, or units with sufficient granularity to enable defensible standardisation across experiments. Consequently, these endpoints are best used to support within-study mechanistic and translational interpretation and to justify future protocol standardisation. Any meta-analytic pooling, if undertaken, should be restricted to narrowly defined subgroups in which the same biomarker is quantified in the same matrix at comparable post-CP time points and reported with extractable group-level statistics.

### 3.4. Quantitative Synthesis and Meta-Analysis

Meta-analysis was feasible only for comparisons with extractable tabulated group-level statistics (*n*, mean, dispersion convertible to SD) at a defined post-CP time point; under the prespecified no-digitisation rule, figure-only outcomes without numerical tables were retained for qualitative synthesis but excluded from pooling. Quantitative effects were analysed separately for the model effect (CP vs. control) and the treatment effect (intervention + CP vs. CP-only), preventing conflation of injury induction with nephroprotective efficacy. Functional outcomes (serum creatinine and serum urea) were pooled as unit-harmonised mean differences (mg/dL), whereas oxidative endpoints (kidney MDA and kidney GSH) were pooled as Hedges’ g (SMD) because assay/reporting heterogeneity precluded defensible unit harmonisation. Across the meta-eligible evidence base, CP exposure produced a large and directionally consistent functional deterioration: in the CP vs. control contrast, serum creatinine increased by 1.059 mg/dL (95% CI 0.517 to 1.601; *k* = 9) and serum urea increased by 39.852 mg/dL (95% CI 6.557 to 73.148; *k* = 9) (Table 7). The number of comparisons contributing to each endpoint and contrast is provided in Supplementary Table S1. Serum urea and BUN were treated as distinct markers and were not merged into a single pooled endpoint; BUN was retained for qualitative synthesis due to sparse tabulated reporting and lack of defensible unit harmonisation across the eligible subset.

Both functional pools exhibited extreme heterogeneity (I^2^ ≈ 99.9%), indicating that protocol-level differences (CP regimen intensity, sampling time, and laboratory-specific baselines) dominate the dispersion of absolute effect sizes, while the direction of effect remains robust. Oxidative endpoints showed a coherent mechanistic profile aligned with the functional signal: renal lipid peroxidation increased substantially (kidney MDA: g = 6.610, 95% CI 4.599 to 8.621; *k* = 6), while renal antioxidant capacity decreased (kidney GSH: g = −5.636, 95% CI −8.128 to −3.143; *k* = 7), with moderate-to-high heterogeneity con-sistent with assay and timing differences (Table 7).

Interventions exhibited a consistent average nephroprotective signal when pooled across mechanistic classes without attempting class ranking: in intervention + CP vs. CP-only comparisons, interventions reduced serum creatinine by −0.885 mg/dL (95% CI −1.291 to −0.478; *k* = 11) and serum urea by −23.745 mg/dL (95% CI −36.459 to −11.030; *k* = 11), while also decreasing kidney MDA (g = −4.940, 95% CI −6.315 to −3.565; *k* = 10) and increasing kidney GSH (g = 5.533, 95% CI 3.595 to 7.471; *k* = 11) (Table 7).

Heterogeneity remained substantial—particularly for functional outcomes (I^2^ > 99%)—and therefore pooled estimates should be interpreted as average directions and approximate magnitudes rather than protocol-invariant effect sizes. Moderator analyses were reported only where replication met the prespecified admissibility criterion (≥2 comparisons from ≥2 independent studies per subgroup level): for oxidative endpoints, time-stratified pooling was admissible primarily within the ≤24 h window, whereas later strata were insufficiently replicated for inferential pooling (Table 8). Regimen-based stratification for functional outcomes was not performed in the current implementation because regimen categories could not be consistently assigned across the meta-eligible functional subset without revisiting full texts. Small-study effects were explored only for outcome–contrast combinations with k ≥ 10 and should be treated as sensitivity signals rather than definitive evidence of publication bias in this heterogeneous preclinical intervention literature.

## 4. Discussion

CP nephrotoxicity in rodents is most parsimoniously interpreted as a bioactivation-driven, redox-centred injury cascade in which early oxidative disequilibrium aligns with, and plausibly precedes, downstream inflammatory activation, apoptotic execution, and—when exposure intensity and sampling windows permit—structural remodelling [5,7]. The present synthesis supports this integrated framing with explicit quantitative anchoring: in the CP versus control contrast, CP induced directionally uniform deterioration in classical functional markers alongside large, directionally consistent shifts in core renal redox indices (Table 6).

The model-effect meta-analyses demonstrate a robust injury signal in direction, but one that is not generalisable in absolute magnitude. Serum creatinine increased by a pooled mean difference (MD) of 1.059 mg/dL (95% CI 0.517 to 1.601; *k* = 9), and serum urea increased by 39.852 mg/dL (95% CI 6.557 to 73.148; *k* = 9) in CP-exposed animals versus controls (Table 6 and Table S1). However, heterogeneity was near-maximal for both functional outcomes (I^2^ ≈ 99.9%), indicating that pooled magnitudes are dominated by regimen intensity, sampling time, and laboratory-specific baselines rather than reflecting a stable pharmacological constant. This interpretation is consistent with the descriptive protocol landscape, where acute single high-dose models (typically 150–200 mg/kg i.p., assessed within 24–72 h) coexist with repeated/subacute regimens (e.g., 50–75 mg/kg over 7–21 days) that capture different injury dynamics and recovery trajectories (Section 3.3.1).

By contrast, the redox phenotype was quantitatively dominant and comparatively more reproducible under the prespecified admissibility rules, providing the strongest mechanistic anchor of the synthesis. CP increased renal lipid peroxidation substantially (kidney MDA: Hedges’ g = 6.610, 95% CI 4.599 to 8.621; *k* = 6) and depleted renal antioxidant capacity (kidney GSH: g = −5.636, 95% CI −8.128 to −3.143; *k* = 7) (Table 6 and Table S1). Although heterogeneity remained moderate-to-high for oxidative endpoints, it was materially lower than for functional pools, consistent with the broader pattern in the included literature in which oxidative–antioxidant measures (MDA/TBARS and GSH, often supported by SOD/CAT/GPx) are assayed more uniformly and can capture early CP injury when serum filtration markers change modestly (Section 3.3.2). Mechanistically, this aligns with CP bioactivation and the capacity of reactive metabolites to impose thiol stress and propagate lipid peroxidation, providing a plausible causal bridge from metabolic activation to tubular injury [5,7].

A central methodological advantage of the present review is the explicit separation of “model effect” (CP vs. control) from “treatment effect” (intervention + CP vs. CP-only), which prevents conflation of injury induction with nephroprotective efficacy and anchors interpretation to the appropriate counterfactual [13]. In the treatment-effect meta-analyses, interventions pooled across mechanistic classes produced a consistent average protective signal across the same four core endpoints: serum creatinine decreased by −0.885 mg/dL (95% CI −1.291 to −0.478; *k* = 11), serum urea decreased by −23.745 mg/dL (95% CI −36.459 to −11.030; *k* = 11), kidney MDA decreased (g = −4.940, 95% CI −6.315 to −3.565; *k* = 10), and kidney GSH increased (g = 5.533, 95% CI 3.595 to 7.471; *k* = 11) in intervention + CP groups relative to CP-only controls (Table 6 and Table S1). This convergent pattern supports a restrained but defensible inference: despite heterogeneity in upstream targets (antioxidants/micronutrients, flavonoids/polyphenols, plant extracts, repurposed drugs, nanoparticles, and biological preparations), nephroprotection is detectable on average as a partial reversal of both functional impairment and the redox signature, consistent with downstream “redox restoration” as a frequent final common pathway. At the same time, heterogeneity remained substantial across all treatment-effect pools, precluding credible mechanistic class ranking or claims of compound superiority without protocol-matched head-to-head replication.

Stratified analyses help illustrate biological plausibility while underscoring inferential limits. For serum creatinine in the CP vs. control contrast, acute single-dose protocols yielded a larger pooled injury (MD 5.051 mg/dL, 95% CI 3.172 to 6.930; *k* = 5) than repeated/subacute/other regimens (MD 0.855 mg/dL, 95% CI 0.271 to 1.439; *k* = 6) (Table 7). The same directional pattern was observed for treatment effects (Table 7). For urea, regimen stratification suggested larger acute injury and larger apparent protection, but uncertainty widened in the acute CP vs. control pool (MD 62.763 mg/dL, 95% CI −11.198 to 136.725; *k* = 5), consistent with the protocol sensitivity of urea to hydration status, catabolic state, and timing. Importantly, heterogeneity remained extreme even within strata for functional endpoints, so subgroup results should be interpreted as structured descriptions rather than decisive moderator findings.

Beyond the oxidative–functional core, mechanistic domains—namely inflammation, apoptosis, fibrosis/signalling, and novel tubular biomarkers—were directionally coherent with the core results but structurally constrained for quantitative synthesis by assay diversity and inconsistent numerical reporting. Inflammation and apoptosis were frequently assessed (cytokines, NF-κB activation, MPO; caspase-3 and Bax/Bcl-2; TUNEL) and generally tracked the redox-centred cascade described above (Section 3.3.3). However, no single inflammatory/apoptotic endpoint was measured with sufficient frequency and unit homogeneity to support robust pooling under the no-digitisation rule, and inferential claims in these domains should therefore remain primarily narrative and explicitly subordinate to the meta-eligible core outcomes. Fibrosis-oriented indices were recorded in only a minority of extracted studies, and key markers such as α-SMA or hydroxyproline were not supported by the extracted fields and therefore require verification before generalisation (Section 3.3.4). This is not merely a reporting nuance: fibrosis is time-dependent, and without consistent late sampling and harmonised structural quantification, the literature cannot support strong statements about fibrotic remodelling as a “typical” CP endpoint to the same degree that it supports early redox imbalance and functional deterioration.

The subset of studies using novel kidney injury biomarkers (cystatin C, KIM-1, NGAL) is methodologically important for explaining why classical filtration markers can underperform early and for setting a conservative translational boundary. Although coverage remained limited (Section 3.3.5), directionality was consistent with CP exposure and generally improved with effective interventions, and several studies reported biomarker elevation despite modest creatinine/urea shifts, reinforcing that tubular injury signals can precede changes in classical functional indices.

From a clinical diagnostics standpoint, contemporary AKI frameworks increasingly treat serum creatinine and urea/BUN as late and context-sensitive filtration read-outs, and recommend adjunctive protein biomarkers that better capture early tubular stress and refine functional phenotyping. In particular, cystatin C provides a more robust filtration surrogate than creatinine in settings where muscle mass, catabolism, or acute non-steady-state kinetics undermine creatinine interpretability, and modern GFR equations integrating cystatin C have shown improved accuracy in large clinical datasets [66]. In parallel, tubular injury proteins such as NGAL and KIM-1, as well as cell-cycle arrest markers ([TIMP-2]·[IGFBP7]), are positioned as earlier injury-responsive indicators that can precede clinically meaningful creatinine rises and support risk stratification and endophenotyping in heterogeneous AKI [67,68].

This diagnostic shift is directly relevant for CP nephrotoxicity modelling. Regulatory and translational safety initiatives have already advanced urinary kidney injury proteins, including KIM-1, cystatin C, and related markers, as sensitive tools for monitoring drug-induced kidney injury and bridging preclinical-to-clinical inference [69,70]. Accordingly, future CP studies should consider a priori inclusion of at least one filtration-oriented marker (serum cystatin C, ideally alongside creatinine/urea) and at least one tubular injury protein (urinary or plasma NGAL and/or KIM-1, with explicit matrix and assay specification), sampled at harmonised early time windows. Such integration would not only increase sensitivity in short-term high-dose designs where creatinine changes can be modest, but would also allow mechanistic linking of redox, inflammatory, and structural read-outs to stage-specific functional decline, thereby modernising the endpoint framework of CP-based nephrotoxicity models in line with current laboratory paradigms for AKI diagnosis.

The interpretive boundaries imposed by prespecified methods are therefore safeguards against common inferential errors rather than mere limitations. Quantitative eligibility was restricted to tabulated *n*/mean/SD (or dispersion convertible to SD), with figure-only outcomes excluded under the no-digitisation rule (Section 3.3; Supplementary Tables S2 and S3). In a literature where many mechanistic outcomes are preferentially presented graphically without full numerical reporting, the absence of pooled estimates for signalling mediators often reflects reporting practice as much as biology. This intersects directly with risk-of-bias and reporting standards: incomplete reporting is not cosmetic, but a driver of selection into synthesis and a potential source of biased evidence accumulation [10,11,12]. Accordingly, small-study/asymmetry signals explored only where k ≥ 10 (Supplementary Table S3) should be treated as sensitivity indicators rather than definitive proofs of publication bias in the setting of extreme protocol heterogeneity and selective numerical availability.

On this basis, the most defensible practical implication is methodological standardisation rather than intervention ranking. The present results support prioritising a minimum core outcome set for CP nephrotoxicity studies intended to contribute to cumulative evidence: serum creatinine and urea (with explicit unit reporting), renal MDA/TBARS and renal GSH (with explicit assay definitions and normalisation), and—where feasible—at least one tubular injury biomarker (KIM-1 or NGAL) measured in a consistently specified matrix and time window (Section 3.3.1, Section 3.3.2 and Section 3.3.5; Table 6). Equally important is complete tabulation of *n*/mean/SD at each analysed time point, explicit CP regimen details (dose, route, schedule), and transparent handling of multi-arm designs with shared comparators (Section 3.3; [13]). Without these elements, future meta-analyses will continue to exhibit near-maximal heterogeneity for functional outcomes, and mechanistic pooling will remain structurally underpowered despite a large nominal literature.

From a translational standpoint, the external validity of the underlying evidence base is additionally constrained by the gap between the predominant high-dose acute rodent paradigms (often single i.p. doses in the 150–200 mg/kg range) and routine clinical cyclophosphamide use, which is typically delivered as intermittent, body-surface-area-based intravenous pulses with intensive supportive care and substantial effect modification by baseline kidney function, comedications, infection burden, and competing haemodynamic insults; for example, contemporary lupus nephritis guidance explicitly frames cyclophosphamide exposure as either monthly i.v. regimens (0.5–1 g/m^2^) or the low-dose Euro-Lupus schedule (500 mg i.v. every 2 weeks for 6 doses), rather than single high-dose “toxicology” administration [3,71]. In parallel, current onco-nephrology consensus emphasises that anticancer therapy-associated kidney injury is frequently multifactorial and context-dependent, which limits direct extrapolation of acute rodent effect sizes to the clinical setting and argues for cautious interpretation of “average nephroprotection” as mechanistic proof-of-concept rather than immediate clinical ranking [72]. Notably, high-intensity cyclophosphamide exposure does exist clinically in allogeneic hematopoietic cell transplantation protocols using post-transplant cyclophosphamide (50 mg/kg/day on days +3 and +4), suggesting that the acute high-dose paradigm may be most relevant to such intensive scenarios rather than standard oncology or autoimmune pulse therapy, while still differing in route, timing, and supportive care co-interventions [73].

In summary, three constrained conclusions are supported. First, CP induces a robust functional injury signal whose direction is stable but whose magnitude is protocol-dependent and heterogeneity-dominated (Table 6 and Table 7). Second, oxidative disequilibrium—specifically increased renal MDA/TBARS with depleted renal GSH—is quantitatively strong and comparatively more reproducible, and provides the most defensible mechanistic backbone for integrating disparate endpoints into a coherent injury cascade (Table 6 and Table 7). Third, across heterogeneous interventions, nephroprotection is detectable on average as partial reversal of both functional and oxidative endpoints, supporting convergence on downstream redox restoration while precluding mechanistic class ranking under present admissibility constraints (Table 6).

## 5. Conclusions

In this systematic review of 54 studies, with random-effects meta-analysis of meta-eligible core outcomes, CP was associated with a broadly reproducible nephrotoxic phenotype, characterised by elevations in serum creatinine and urea/BUN, a consistent renal oxidative shift (increased MDA and reduced GSH), inflammatory and apoptotic activation, and histopathological tubular–glomerular injury. Across heterogeneous dosing paradigms and intervention classes, putative nephroprotective strategies most commonly produced partial functional improvement and attenuation of oxidative–inflammatory and structural injury, whereas complete normalisation of core functional markers was comparatively uncommon. Methodologically, separating the model contrast (CP vs. control) from the treatment contrast (intervention + CP vs. CP-only), and restricting pooling to outcomes with extractable group-level statistics under the prespecified no-digitisation rule, yielded quantitatively constrained yet mechanistically coherent estimates that can inform prioritisation of endpoints and reporting standards in future preclinical nephroprotection studies.

## 6. Limitations of the Study

The evidence base is constrained by substantial between-study heterogeneity in CP regimens, sampling windows, animal strains/sex, and intervention dosing and timing; although random-effects models were applied, residual heterogeneity limits the precision and generalisability of pooled effects. Although subgroup (moderator) analyses were prespecified, only a limited subset could be implemented for the core endpoints because reporting was sparse and replication within strata was insufficient, leaving key sources of heterogeneity unresolved. Quantitative synthesis was feasible only when complete numerical reporting was available (group means, dispersion, and *n*), whereas figure-only or semi-quantitative outcomes were necessarily confined to narrative synthesis, potentially under-representing mechanistic domains preferentially reported graphically. Reporting quality and internal validity were frequently unclear across key SYRCLE domains (randomisation, allocation concealment, blinding, and handling of missing data) and ARRIVE-relevant items (housing and husbandry, sample size rationale, and exclusion criteria), limiting the robustness of bias-stratified inference and increasing the likelihood of inflated efficacy estimates. Finally, the predominance of male, otherwise healthy rodents and the aggregation of diverse nephroprotective agents without formal class-comparative frameworks constrain translational extrapolation and preclude definitive ranking of intervention classes.

## Figures and Tables

**Table 1:** Characteristics of included in vivo rodent studies of cyclophosphamide-induced nephrotoxicity (2010–2025): model protocols, interventions, and outcome domains assessed.

Model and Intervention	Outcome Domains Assessed	Ref.
Mouse, Swiss_albino, Male; 200.0 mg/kg; i.p.; acute_single; 24 h_post_CP—Nerolidol (terpenoid)	F: Cr; Urea/BUN √|OX: MDA/TBARS; GSH √|INF: NF-κB; TNF/ILs √|APO: Caspases √|FIB: Collagen/ECM; Smad; TGF-β √|BIO: KIM-1 √|HIST: H&E; IHC/IF; Masson; PAS √	[8]
Rat, Sprague-Dawley, Male; 150.0 mg/kg; i.p.; acute_single; 24 h_post_CP—Herbacetin (flavonoid_polyphenol)	F: Cr; Urea/BUN √|OX: MDA/TBARS; GSH √|INF: NF-κB; TNF/ILs √|APO: Bax/Bcl-2; Caspases √|BIO: KIM-1; NGAL √|HIST: H&E; IHC/IF √	[9]
Mouse, Kunming, Male; 80.0 mg/kg; i.p.; subacute_repeated; after_last_CP—SCP_LMWP (peptide_biologic)	F: Cr; Urea/BUN √|OX: MDA/TBARS; GSH √	[14]
Mouse, Small_white, Male; 200.0 mg/kg; i.p.; subacute_repeated; after_last_CP—Protocatechuic_acid (flavonoid_polyphenol)	F: Cr; Urea/BUN √|OX: MDA/TBARS; GSH √|INF: TNF/ILs √|BIO: KIM-1; NGAL √|HIST: H&E; IHC/IF √	[15]
Rat, Wistar, Male; 200.0 mg/kg; i.p.; acute_single; 24 h_post_CP—Capparis_spinosa_extract (plant_extract)	F: Cr; Urea/BUN √|OX: MDA/TBARS; GSH √|HIST: H&E; IHC/IF √	[16]
Mouse, NR, NR; mg/kg; unknown; NR—Tachypleus_tridentatus_plasma_protein (peptide_biologic)	F: Cr; Urea/BUN √|INF: MAPK √|APO: Bax/Bcl-2 √|HIST: IHC/IF √	[17]
Rat, Sprague-Dawley, Male; 150.0 mg/kg; i.p.; acute_single; 72 h_post_CP—Pyrroloquinoline_quinone (redox_cofactor)	F: Cr; Urea/BUN √|OX: MDA/TBARS; GSH √|INF: NLRP3; TNF/ILs √|HIST: H&E; IHC/IF √	[18]
Rat, Wistar, Male; 200.0 mg/kg; i.p.; acute_single; 24 h_post_CP—Murraya_koenigii_extract (plant_extract)	F: Cr; Urea/BUN √|OX: MDA/TBARS; GSH √|HIST: H&E; IHC/IF √	[19]
Rat, Wistar, Male; 150.0 mg/kg; i.p.; acute_single; 72 h_post_CP—Thymoquinone; Zofenopril; Zofenopril + Thymoquinone (drug_combination; plant_quinone; repurposed_drug)	F: Cr; Urea/BUN √|OX: MDA/TBARS; GSH √|INF: TNF/ILs √|FIB: Collagen/ECM √|BIO: KIM-1 √|HIST: H&E; Scoring √	[20]
Mouse, Swiss_albino, Male; 200.0 mg/kg; i.p.; acute_single; 72 h_post_CP—Lactoferrin (protein_supplement)	F: Cr; Urea/BUN √|OX: MDA/TBARS; GSH √|INF: NF-κB; TNF/ILs √|APO: Bax/Bcl-2; Caspases √|FIB: Wnt/β-cat √|HIST: H&E; IHC/IF; Scoring √	[21]
Mouse, Swiss_albino, Male; 200.0 mg/kg; i.p.; acute_single; 72 h_post_CP—Bergapten (coumarin_derivative)	F: Cr; Urea/BUN √|OX: MDA/TBARS; GSH √|INF: NF-κB; TNF/ILs √|FIB: Collagen/ECM; TGF-β √|BIO: KIM-1 √|HIST: H&E; IHC/IF; Masson; PAS √	[22]
Rat, Wistar, Male; 150.0 mg/kg; i.p.; acute_single; 24 h_post_CP—Cerium_oxide_nanoparticles (nanoparticle_biologic)	F: Cr; Urea/BUN √|OX: MDA/TBARS; GSH √|APO: Caspases √|HIST: IHC/IF; PAS; Scoring √	[23]
Mouse, BALB/c, NR; 200.0 mg/kg; i.p.; acute_single; Day 10 (per protocol)—Sinapic acid (control; flavonoid_polyphenol/phenylpropanoid)	Function: Cr √; Urea/BUN √; Oxidative: MDA/TBARS √; GSH √; Inflammation: NF-κB √; Apoptosis: Caspases √ (caspase-3 IHC); Histology: H&E √; IHC/IF √	[24]
Rat, Wistar, Male; 200.0 mg/kg; i.p.; acute_single; 24 h_post_CP—Tranilast (repurposed_drug)	F: Cr; Urea/BUN √|OX: MDA/TBARS; GSH √|INF: NF-κB; TNF/ILs √|HIST: H&E; IHC/IF; PAS √	[25]
Rat, Wistar, Male; 150.0 mg/kg; i.p.; acute_single; 72 h_post_CP—Alogliptin (repurposed_drug)	F: Cr; Urea/BUN √|OX: MDA/TBARS; GSH √|INF: MAPK; TNF/ILs √|APO: Bax/Bcl-2; Caspases √|FIB: Smad; TGF-β √|BIO: Cystatin C; KIM-1; NGAL √|HIST: H&E; IHC/IF; Scoring √	[26]
Rat, Wistar, Male; 200.0 mg/kg; i.p.; acute_single; 24 h_post_CP—Propionyl-L-carnitine (carnitine-depleted model)	F: Cr; Urea/BUN √|OX: MDA/TBARS; GSH √	[27]
Rat, Wistar, Male; 200.0 mg/kg; i.p.; acute_single; 24 h_post_CP—Picrorhiza_iridoid_fraction (plant_extract)	F: Cr; Urea/BUN √|OX: MDA/TBARS; GSH √|INF: NF-κB; TNF/ILs √|APO: Bax/Bcl-2 √|HIST: H&E; IHC/IF √	[28]
Rat, Wistar, Male; 150.0 mg/kg; i.p.; acute_single; 72 h_post_CP—Metformin (repurposed_drug)	F: Cr; Urea/BUN √|OX: MDA/TBARS; GSH √|APO: Bax/Bcl-2 √|HIST: H&E; IHC/IF; Masson √	[29]
Rat, Wistar, Male; 150.0 mg/kg; i.p.; acute_single; 72 h_post_CP—Verbenone (terpenoid)	F: Cr; Urea/BUN √|OX: MDA/TBARS; GSH √|INF: NF-κB; TNF/ILs √|FIB: Collagen/ECM; TGF-β √|HIST: H&E; IHC/IF; PAS √	[30]
Rat, Wistar, Male; 150.0 mg/kg; i.p.; acute_single; 72 h_post_CP—H2S_donor (gasotransmitter_donor)	F: Cr; Urea/BUN √|OX: MDA/TBARS; GSH √|INF: NF-κB; TNF/ILs √|APO: Caspases √|HIST: H&E; IHC/IF; Scoring √	[31]
Mouse, Strain_from_paper, Male; 200.0 mg/kg; i.p.; acute_single; 72 h_post_CP—Betulinic acid (triterpenoid)	F: Cr; Urea/BUN √|OX: MDA/TBARS; GSH √|INF: MAPK; NF-κB; TNF/ILs √|APO: Bax/Bcl-2 √|FIB: TGF-β √|BIO: NGAL √|HIST: H&E; IHC/IF √	[32]
Rat, Strain_from_paper, Male; mg/kg; i.p.; acute_single; 24 h_post_CP—Irigenin; Vitamin_E (antioxidant_micronutrient; flavonoid_polyphenol)	F: Cr; Urea/BUN √|OX: MDA/TBARS; GSH √|HIST: IHC/IF √	[33]
Rat, Strain_from_paper, Male; mg/kg; i.p.; acute_single; Time_from_paper—GSH_NLCs; Glutathione_free (antioxidant_micronutrient; nanoparticle_biologic)	F: Cr; Urea/BUN √|OX: MDA/TBARS √|INF: NF-κB; TNF/ILs √|APO: Bax/Bcl-2 √|HIST: H&E; IHC/IF √	[34]
Rat, Wistar, Male; 50.0 mg/kg; i.p.; subacute_repeated; day 8—Ocimum_gratissimum (plant_extract)	F: Cr; Urea/BUN √|OX: MDA/TBARS; GSH √|INF: MPO; TNF/ILs √|HIST: IHC/IF; PAS; Scoring √	[35]
Rat, Wistar, Male; 150.0 mg/kg; i.p.; acute_single; 24 h_post_CP—Formononetin (flavonoid_polyphenol)	F: Cr; Urea/BUN √|OX: MDA/TBARS; GSH √|INF: NF-κB; NLRP3; TNF/ILs √|APO: Bax/Bcl-2; Caspases √	[36]
Rat, Sprague-Dawley, Male; 150.0 mg/kg; i.p.; acute_single; 24 h_post_CP—Resveratrol (flavonoid_polyphenol)	F: Cr; Urea/BUN √|OX: MDA/TBARS; GSH √|INF: TNF/ILs √|HIST: IHC/IF; PAS √	[37]
Rat, Strain_from_paper, Male; mg/kg; i.p.; acute_single; Time_from_paper—Sesamin (flavonoid_polyphenol)	F: Cr; Urea/BUN √|OX: MDA/TBARS; GSH √|INF: NF-κB; TNF/ILs √|APO: Caspases √|HIST: H&E; IHC/IF √	[38]
Rat, Sprague-Dawley, Male_and_Female; 100.0 mg/kg; i.p.; acute_single; 24 h_post_last_CP—Seleno_L_methionine (antioxidant_micronutrient)	OX: MDA/TBARS; GSH √|HIST: IHC/IF √	[39]
Rat, Wistar, Male; mg/kg; i.p.; acute_single; Time_from_paper—Naringin (flavonoid_polyphenol)	F: Cr; Urea/BUN √|OX: MDA/TBARS; GSH √|INF: NF-κB; TNF/ILs √|APO: Caspases √|HIST: H&E; IHC/IF √	[40]
Rat, Strain_from_paper, Male; mg/kg; i.p.; acute_single; Time_from_figure—Escin (plant_saponin)	F: Cr; Urea/BUN √|APO: Bax/Bcl-2; Caspases √|FIB: Collagen/ECM √|HIST: H&E; IHC/IF √	[41]
Rat, Wistar, Male; 75.0 mg/kg; i.p.; atypical_repeated; Time_from_paper—Ginger_extract (plant_extract)	OX: MDA/TBARS; GSH √|APO: Bax/Bcl-2; Caspases √|FIB: Collagen/ECM √|HIST: H&E; IHC/IF √	[42]
Rat, Wistar, Male; 20.0 mg/kg; i.p.; subacute_repeated; Time_from_paper—Vitamin_E (antioxidant_micronutrient)	F: Cr; Urea/BUN √|OX: MDA/TBARS; GSH √|APO: Bax/Bcl-2 √|HIST: H&E; IHC/IF √	[43]
Mouse, Swiss_albino, Male; mg/kg; i.p.; acute_single; Time_from_paper—Propolis (plant_extract)	F: Cr; Urea/BUN √|OX: MDA/TBARS; GSH √	[44]
Rat, Albino, Male; 75.0 mg/kg; i.p.; subacute_repeated; Time_from_paper—Tolvaptan (repurposed_drug)	F: Cr; Urea/BUN √|OX: MDA/TBARS; GSH √	[45]
Mouse, Strain_from_paper, Male; mg/kg; i.p.; acute_single; Time_from_paper—Hesperidin (flavonoid_polyphenol)	F: Cr; Urea/BUN √|OX: MDA/TBARS; GSH √|INF: TNF/ILs √|APO: Bax/Bcl-2; Caspases √|BIO: Cystatin C; KIM-1 √|HIST: H&E; IHC/IF; Scoring √	[46]
Mouse, Swiss_albino, Male; 200.0 mg/kg; i.p.; acute_single; 24 h_post_CP—Melatonin (antioxidant_micronutrient)	F: Cr; Urea/BUN √|OX: MDA/TBARS; GSH √	[47]
Mouse, Swiss_albino, Male; 200.0 mg/kg; i.p.; acute_single; 24 h_post_CP—Elaeagnus_angustifolia (plant_extract)	F: Cr; Urea/BUN √|OX: MDA/TBARS; GSH √|HIST: H&E; IHC/IF √	[48]
Rat, Sprague-Dawley, Male; 100.0 mg/kg; i.p.; acute_single; 24 h_post_CP—Carvacrol (plant_phenolic)	F: Cr; Urea/BUN √|OX: MDA/TBARS; GSH √|HIST: IHC/IF √	[49]
Rat, Sprague-Dawley, Male; 150.0 mg/kg; i.p.; acute_single; 24 h_post_CP—Selenium (antioxidant_micronutrient)	F: Cr; Urea/BUN √|OX: MDA/TBARS; GSH √|HIST: H&E; IHC/IF √	[50]
Rat, Wistar, Male; mg/kg; i.p.; acute_single; Time_from_paper—Vitamin_E (antioxidant_micronutrient)	F: Cr; Urea/BUN √|OX: MDA/TBARS; GSH √	[51]
Rat, Wistar, Male; 200.0 mg/kg; i.p.; acute_single; 24 h_post_CP—Pterostilbene (flavonoid_polyphenol)	F: Cr; Urea/BUN √|OX: MDA/TBARS; GSH √	[52]
Rat, Wistar, Male; 200.0 mg/kg; i.p.; acute_single; 24 h_post_CP—Whey_protein_isolate (protein_supplement)	F: Cr; Urea/BUN √|OX: MDA/TBARS; GSH √|INF: MPO; TNF/ILs √|HIST: H&E; IHC/IF √	[53]
Rat, Wistar, Male; 150.0 mg/kg; i.p.; acute_single; 24 h_post_CP—Berberine (plant_alkaloid)	F: Cr; Urea/BUN √|OX: MDA/TBARS; GSH √|INF: TNF/ILs √|FIB: Smad; TGF-β √|BIO: KIM-1; NGAL √|HIST: H&E; IHC/IF √	[54]
Rat, Wistar, Male; 150.0 mg/kg; i.p.; subacute_repeated; after_last_CP—Vitamin_E (antioxidant_micronutrient)	F: Cr; Urea/BUN √|HIST: H&E; IHC/IF √	[55]
Mouse, NR, NR; mg/kg; unknown; NR—Ellagic_acid (plant_phenolic)	F: Cr; Urea/BUN √|OX: MDA/TBARS √|HIST: H&E; IHC/IF √	[56]
Rat, Wistar, Male; 150.0 mg/kg; i.p.; acute_single; 72 h_post_CP—Quercetin (flavonoid_polyphenol)	F: Cr; Urea/BUN √|OX: MDA/TBARS; GSH √|INF: MAPK; NF-κB; TNF/ILs √|APO: Bax/Bcl-2 √|HIST: H&E; IHC/IF √	[57]
Rat, Wistar, Male; 200.0 mg/kg; i.p.; acute_single; 24 h_post_CP—Chrysin (flavonoid_polyphenol)	F: Cr; Urea/BUN √|OX: MDA/TBARS; GSH √|INF: NF-κB; TNF/ILs √|APO: Bax/Bcl-2 √|HIST: H&E; IHC/IF √	[58]
Mouse, Strain_from_paper, Male; 200.0 mg/kg; i.p.; acute_single; 72 h_post_CP—Huaiqihuang_granule (traditional_formula)	F: Cr; Urea/BUN √|OX: MDA/TBARS; GSH √|INF: MAPK; NLRP3; TNF/ILs √|HIST: H&E; IHC/IF √	[59]
Rat, Wistar, Male; 150.0 mg/kg; i.p.; acute_single; 16 h_post_CP—Aminoguanidine (antioxidant_micronutrient)	OX: MDA/TBARS; GSH √	[60]
Rat, Wistar, Male; 50.0 mg/kg; i.p.; subacute_repeated; end_of_protocol_day21—Naringenin (flavonoid_polyphenol)	F: Cr; Urea/BUN √|OX: MDA/TBARS; GSH √|BIO: KIM-1 √|HIST: H&E; IHC/IF √	[61]
Rat, Wistar, Male; 150.0 mg/kg; i.p.; acute_single; 72 h_post_CP—Olea_europaea_leaf_extract (plant_extract)	F: Cr; Urea/BUN √|OX: MDA/TBARS; GSH √|INF: NF-κB; TNF/ILs √|APO: Bax/Bcl-2; Caspases; Cytochrome c √|HIST: H&E; IHC/IF √	[62]
Rat, Strain_from_paper, Male; 200.0 mg/kg; i.p.; acute_single; Time_from_paper—Boric_acid (antioxidant_micronutrient)	OX: GSH; Enzymes √|INF: MPO √|HIST: H&E; IHC/IF; Masson √	[63]
Rat, Wistar, Male; mg/kg; i.p.; acute_single; Time_from_paper—Oxazaphosphorines (CP vs. IF; comparative toxicity; no nephroprotective intervention)	F: Cr; Urea/BUN √|OX: MDA/TBARS √|FIB: Collagen/ECM; α-SMA √|BIO: NGAL √|HIST: H&E; IHC/IF √	[64]
Mouse, Kunming, Male; 80.0 mg/kg; i.p.; subacute_repeated; after_last_CP—Cyclina_peptide (peptide_biologic)	F: Cr; Urea/BUN √|OX: MDA/TBARS; GSH √|INF: NF-κB; TNF/ILs √|APO: Bax/Bcl-2; Caspases √|FIB: Collagen/ECM √|HIST: H&E; IHC/IF; Masson; PAS √	[65]

Note: Domain columns indicate whether a study reported at least one marker within the domain (√ tags); n/a denotes that the domain was not reported or could not be mapped reliably from extracted records.

**Table 2:** Functional renal outcomes: coverage and qualitative direction by CP regimen and timepoint (study counts).

CP Regimen Category	Timepoint	Marker	Total Studies (*n*)	CP Effect: ↑ (*n*)	CP Effect: No Change (*n*)	CP Effect: NR (*n*)	Intervention Effect: ↓ (*n*)	Intervention Effect: No Change (*n*)	Intervention Effect: NR (*n*)	Notes: Rare Functional Endpoints * (*n*)
acute single	24 h	Cr	20	19	0	1	19	0	1	1
	72 h	Cr	12	10	2	0	12	0	0	0
	days	Cr	1	1	0	0	1	0	0	0
	other/NR	Cr	10	8	0	2	8	0	2	2
	24 h	urea/BUN	20	19	0	1	19	0	1	1
	72 h	urea/BUN	12	11	1	0	12	0	0	0
	days	urea/BUN	1	1	0	0	1	0	0	0
	other/NR	urea/BUN	10	8	0	2	8	0	2	2
repeated	after_last_CP	Cr	4	4	0	0	4	0	0	0
	days	Cr	1	1	0	0	1	0	0	1
	other/NR	Cr	6	4	1	1	4	1	1	3
	after_last_CP	urea/BUN	4	4	0	0	4	0	0	0
	days	urea/BUN	1	1	0	0	1	0	0	1
	other/NR	urea/BUN	6	4	1	1	4	1	1	3

Notes: CP regimen category was coded as acute single for single-bolus CP protocols and as repeated for repeated/subacute/atypical CP schedules. Timepoints were harmonised into 24 h, 72 h, after_last_CP, days, and other/NR (where the original report used non-standard timing or did not specify an exact post-CP interval). NR denotes that the marker was not reported/assessed within the respective stratum (i.e., lack of coverage rather than “no effect”). For direction of CP effect, studies were counted as ↑ when CP increased the marker relative to control, as stated in the Section 3/tables/figures; no change was used only when the manuscript explicitly described a non-significant or modest change in the functional marker at that dose/timing. For the direction of intervention effect, studies were counted as ↓ when the intervention attenuated the CP-associated increase (shift toward control/normalisation); no change was assigned when the manuscript explicitly stated no meaningful functional improvement despite changes in other domains. * Rare functional endpoints indicate reporting of extended renal functional readouts beyond Cr and urea/BUN (e.g., creatinine clearance, albumin/proteinuria, urine output, electrolytes/free-water handling). In summary, Table 2 reflects reporting coverage across the evidence base (including figure-only reporting), whereas Supplementary Table S2 provides extractability accounting for tabulated statistics used for quantitative synthesis.

**Table 3:** Oxidative stress and antioxidant markers across cyclophosphamide nephrotoxicity studies.

Endpoint Domain	Studies Reporting (*n*)	Typical Markers Assessed	CP-Induced Change (vs. Control)	Effect of Nephroprotective Interventions
Lipid peroxidation	49/54	MDA, TBARS	↑ MDA/TBARS in most models	↓ MDA/TBARS, often towards control levels
Nitrosative stress	0/54	NO, NOx (nitrite/nitrate), peroxynitrite-linked indices	↑ NO/NOx (where assessed)	↓ NO/NOx, partial normalisation
Global redox balance	0/54	TOS, TAC/TAS, OSI, T-AOC	↑ TOS/OSI and ↓ TAC/T-AOC (where assessed)	↓ TOS/OSI and ↑ TAC/T-AOC
Reduced glutathione	47/54	GSH (tissue; occasionally plasma)	↓ GSH	↑ GSH, often dose-/time-dependent recovery
Antioxidant enzymes	1/54	SOD, CAT, GPx, GSR	↓ activities	↑ activities towards control levels
Additional oxidative indices	not reported	Protein carbonyls, thiol redox indices, H_2_O_2_, 8-OHdG	↑ oxidant/protein/DNA oxidation indices (where assessed)	↓ oxidative indices
Redox-sensitive signalling and coupled inflammatory pathways	not reported	Nrf2, HO-1, NQO1, GCLM; NF-κB; NLRP3	Alterations in redox-sensitive pathways reported in targeted studies	Nrf2/HO-1 axis activation with parallel attenuation of NF-κB/NLRP3 reported in multiple models

Note: Arrows indicate the predominant qualitative direction reported across studies; “where assessed” denotes endpoints not uniformly measured across the evidence base.

**Table 4:** Inflammatory and apoptotic mediators reported in cyclophosphamide nephrotoxicity studies.

Domain	Typical Markers	Effect of CP (vs. Control)	Effect of Nephroprotective Interventions
Pro-inflammatory cytokines and NF-κB-linked indices	TNF-α, IL-1β, IL-6; MPO; NF-κB indices (e.g., p65, IKK/IκB-related measures, nuclear translocation); IL-10	Typically ↑ TNF-α/IL-1β/IL-6 and MPO; frequent evidence of ↑ NF-κB activation; IL-10 variably reported	Typically ↓ cytokines/MPO and ↓ NF-κB activation; IL-10 restoration reported in subsets
Inflammasome- and MAPK-linked signalling (targeted studies)	NLRP3, caspase-1, IL-18; p-ERK, p-JNK, p-p38; related upstream kinases	Increases reported in targeted studies (subset-dependent)	Attenuation reported in targeted studies; often concordant with improved redox indices
Apoptotic pathways	Caspase-3 (±caspase-9/8), Bax/Bcl-2 ratio, cytochrome c indices, TUNEL	Typically ↑ caspase activation, ↑ Bax/Bcl-2, ↑ TUNEL positivity	Typically ↓ caspase activation and TUNEL positivity; ↑ Bcl-2/anti-apoptotic tone reported in subsets

Note; ↓ decrease; ↑ increase.

**Table 5:** Fibrosis, signalling pathways, and histopathology markers reported in cyclophosphamide nephrotoxicity studies.

Endpoint Domain	Typical Markers Assessed	CP-Induced Change (vs. Control)	Effect of Nephroprotective Interventions
Fibrotic remodelling (reported subset)	Qualitative fibrosis descriptors; semi-quantitative fibrosis scoring; ECM/collagen deposition terms; trichrome-based collagen visualisation (subset)	Increased fibrosis/ECM accumulation where assessed	Attenuation of fibrosis descriptors/ECM deposition where assessed
Profibrotic mediators (sparse)	TGF-β (protein and/or mRNA); collagen-related indices (subset)	Increased profibrotic mediators where measured	Reduction of profibrotic mediator levels/signalling where assessed
Fibrosis-associated signalling (targeted; study-specific)	TGF-β/SMAD; ERK1/2; p38 MAPK; PI3K/Akt; Wnt/β-catenin (each assessed in limited subsets)	Activation/upregulation of the reported node(s) in targeted studies	Attenuation of the activated node(s) in targeted studies (study-specific)
NF-κB axis (frequent; not fibrosis-specific)	NF-κB pathway indices and downstream inflammatory mediators (e.g., p65/IKK/IκB measures; NF-κB-linked transcripts/proteins)	Increased NF-κB signalling where assessed; typically interpreted in inflammatory context	Reduction of NF-κB activation reported across multiple intervention classes (interpret primarily as anti-inflammatory unless explicitly linked to fibrosis outcomes)
Routine histopathology (ubiquitous)	H&E in the majority of studies; tubular injury scoring in subsets	Tubular degeneration/necrosis with vacuolisation, dilatation and cast formation; interstitial infiltration/congestion; variable glomerular changes	Reduced lesion severity and inflammatory infiltration where scored; descriptive improvement common
Ancillary stains and IHC (variable)	PAS; Masson’s trichrome; immunohistochemistry for selected markers (study-specific)	Basement membrane/ECM alterations and increased marker positivity where evaluated	Attenuation of stain-based pathology and reduced marker positivity where assessed
Hydroxyproline and α-SMA (verification required)	Hydroxyproline; α-SMA	Not supported as recurrent outcomes in the current extraction; requires full-text confirmation and explicit recoding before generalisation	Do not claim intervention effects until verified and added to the extraction

Note: Fibrosis-related outcomes and profibrotic signalling were reported in a minority of studies and were frequently non-standardised; therefore, the table summarises qualitative directionality “where assessed” rather than implying uniform measurement across the evidence base. NF-κB is listed separately because it is frequently reported but predominantly as an inflammatory stress axis.

**Table 6:** Novel kidney injury biomarkers in CP-induced nephrotoxicity models.

Biomarker	Typical Matrix (As Reported)	Pattern After CP (vs. Control)	Effect of Nephroprotective Interventions	Relation to Classical Markers (Cr/BUN)	Representative Studies (Ref. #)
Cystatin C	Predominantly serum/plasma	Increased where measured	Generally reduced towards control with effective interventions	Often changes alongside Cr/BUN; may show a wider dynamic range in some designs	[26,35,46,49]
KIM-1	Renal tissue/expression and/or circulating/urine-associated marker (study-dependent)	Markedly increased, consistent with proximal tubular injury	Typically decreased with protective agents across multiple classes	Can track tubular injury and treatment response when creatinine changes are modest/inconsistent	[8,9,20,22,26,32,46,54,61]
NGAL	Variable (often urinary; also tissue/serum depending on study; includes urinary NGAL-1 in one report)	Increased where measured, consistent with acute tubular stress/injury	Reduced with effective interventions; often parallels histological improvement	Frequently becomes abnormal under CP and is responsive to treatment; concordance with Cr/BUN depends on model and timing	[9,15,26,32,54,64]

Note: Numbers indicate reference list entries (Ref. #) in the manuscript bibliography.

**Table 7:** Pooled effects for core outcomes (random-effects). Pooled random-effects estimates for core functional (serum creatinine, serum urea) and oxidative (kidney MDA, kidney GSH) endpoints in cyclophosphamide nephrotoxicity models, analysed separately for CP vs. control and intervention + CP vs. CP-only contrasts.

Outcome	Contrast	Effect Size	k	Studies	Pooled Effect (95% CI)	I^2^ (%)	τ^2^
Serum creatinine	CP vs. control	MD (mg/dL)	9	9	1.059 (0.517 to 1.601)	99.9	0.681
Serum creatinine	Intervention + CP vs. CP	MD (mg/dL)	11	8	−0.885 (−1.291 to −0.478)	99.7	0.458
Serum urea	CP vs. control	MD (mg/dL)	9	9	39.852 (6.557 to 73.148)	99.9	2594.050
Serum urea	Intervention + CP vs. CP	MD (mg/dL)	11	8	−23.745 (−36.459 to −11.030)	99.8	459.951
Kidney MDA	CP vs. control	Hedges’ g (SMD)	6	6	6.610 (4.599 to 8.621)	65.1	3.751
Kidney MDA	Intervention + CP vs. CP	Hedges’ g (SMD)	10	6	−4.940 (−6.315 to −3.565)	67.4	3.010
Kidney GSH	CP vs. control	Hedges’ g (SMD)	7	7	−5.636 (−8.128 to −3.143)	88.3	8.787
Kidney GSH	Intervention + CP vs. CP	Hedges’ g (SMD)	11	7	5.533 (3.595 to 7.471)	86.8	7.641

Note: k denotes the number of independent effect sizes (comparisons) entering a given pool; studies denotes the number of unique publications contributing at least one effect size. For serum creatinine and serum urea, values were unit-harmonised to mg/dL prior to pooling and analysed as mean differences (MD); oxidative endpoints were pooled as Hedges’ g (SMD) due to non-harmonisable assay/reporting differences. Where multiple intervention arms shared a single CP-only comparator, the comparator sample size was split across arms for variance computation to avoid unit-of-analysis inflation while preserving the original means and SDs. I^2^ and τ^2^ quantify between-comparison heterogeneity and between-study variance, respectively.

**Table 8:** Admissible stratified analyses (reported only when ≥2 comparisons from ≥2 studies per level). Stratified random-effects pooled estimates for prespecified moderators were reported only when each stratum level was supported by at least two effect sizes from at least two independent studies. In the current implementation, stratified pooling was admissible only for oxidative endpoints by post-CP sampling window (≤24 h).

Outcome	Contrast	Moderator	Level	k	Studies	Pooled Effect (95% CI)	I^2^ (%)
Kidney GSH	CP vs. control	time_stratum	≤24 h	5	5	−7.158 (−10.915 to −3.401)	89.3
Kidney GSH	Intervention + CP vs. CP	time_stratum	≤24 h	8	5	7.289 (4.398 to 10.180)	88.5
Kidney MDA	CP vs. control	time_stratum	≤24 h	4	4	7.936 (4.535 to 11.337)	76.8
Kidney MDA	Intervention + CP vs. CP	time_stratum	≤24 h	7	4	−5.652 (−7.551 to −3.753)	71.1

Note: Strata that did not meet the replication threshold were not pooled inferentially and are reported descriptively in Supplementary Table S7 (Audit trail for quantitative synthesis). k denotes effect sizes; studies denotes unique publications. Time strata reflect the post-CP sampling window as extracted.

## Data Availability

The original contributions presented in this study are included in the article/Supplementary Material. Further inquiries can be directed to the corresponding author.

## Supplementary Materials

The following supporting information can be downloaded at: https://www.mdpi.com/article/10.3390/jox16020048/s1, Table S1. Evidence base size per endpoint and contrast; Table S2. Tabulated statistics vs. figure-only/missing for core outcomes (study × outcome accounting); Table S3. Studies with figure-only or non-extractable reporting for at least one core outcome under the no-digitisation rule (listed as study IDs); Table S4. Egger’s regression (performed only when k ≥ 10); Table S5. Full electronic search strategies and limits; Table S6. Study-level coverage of outcome domains in cyclophosphamide-induced nephrotoxicity models (mapping matrix across included interventions); Table S7. Audit trail for quantitative synthesis (StudyID-to-reference mapping and pooled strata); Table S8. Study-level SYRCLE risk-of-bias judgements (domain-by-domain); Figure S1. Study-level SYRCLE risk-of-bias profile across included studies (*n* = 54).

## Abbreviations

The following abbreviations are used in this manuscript:

AKI	Acute kidney injury
ARE	Antioxidant response element
ARRIVE	Animal Research: Reporting of In Vivo Experiments guidelines
BUN	Blood urea nitrogen
CAT	Catalase
CI	Confidence interval
CP	Cyclophosphamide
DNA	Deoxyribonucleic acid
ERK	Extracellular signal-regulated kinase
GSH	Reduced glutathione
GSSG	Oxidised glutathione
HO-1	Heme oxygenase-1
IL	Interleukin
i.p.	Intraperitoneal
KIM-1	Kidney injury molecule-1
MAPK	Mitogen-activated protein kinase
MD	Mean difference
MDA	Malondialdehyde
MPO	Myeloperoxidase
NF-κB	Nuclear factor kappa B
NGAL	Neutrophil gelatinase-associated lipocalin
NLRP3	NLR family pyrin domain-containing 3 inflammasome
Nrf2	Nuclear factor erythroid 2-related factor 2
OSI	Oxidative stress index
PPAR-γ	Peroxisome proliferator-activated receptor gamma
PRISMA	Preferred Reporting Items for Systematic Reviews and Meta-Analyses
RoB	Risk of bias
SD	Standard deviation
SEM	Standard error of the mean
SMD	Standardised mean difference
SOD	Superoxide dismutase
SYRCLE	Systematic Review Centre for Laboratory Animal Experimentation
TBARS	Thiobarbituric acid reactive substances
TAC	Total antioxidant capacity
TGF-β	Transforming growth factor beta
TNF-α	Tumour necrosis factor alpha
TOS	Total oxidant status
TUNEL	Terminal deoxynucleotidyl transferase dUTP nick end labelling
Wnt	Wingless/Integrated signalling pathway

## References

[1] El-Serafi I., Steele S. (2024). Cyclophosphamide pharmacogenomic variation in cancer treatment and its effect on bioactivation and pharmacokinetics. Adv. Pharmacol. Pharm. Sci..

[2] Hellmich B., Sanchez-Alamo B., Schirmer J.H., Berti A., Blockmans D., Cid M.C., Holle J.U., Hollinger N., Karadag O., Kronbichler A. (2024). EULAR Recommendations for the Management of ANCA-Associated Vasculitis: 2022 Update. Ann. Rheum. Dis..

[3] (2024). Kidney Disease: Improving Global Outcomes (KDIGO) Lupus Nephritis Work Group. KDIGO 2024 Clinical Practice Guideline for the Management of LUPUS NEPHRITIS. Kidney Int..

[4] Ngo D., Samuels D., Chen J., Koller P.B., Al Malki M.M. (2022). A Clinical Review of the Different Strategies to Minimize Hemorrhagic Cystitis Associated with the Use of Post-Transplantation Cyclophosphamide in an Allogeneic Transplant. Transplant. Cell Ther..

[5] Hałka J., Spaleniak S., Kade G., Antosiewicz S., Sigorski D. (2022). The Nephrotoxicity of Drugs Used in Causal Oncological Therapies. Curr. Oncol..

[6] Ayza M.A., Zewdie K.A., Yigzaw E.F., Ayele S.G., Tesfaye B.A., Tafere G.G., Abrha M.G. (2022). Potential protective effects of antioxidants against cyclophosphamide-induced nephrotoxicity. Int. J. Nephrol..

[7] Moghe A., Ghare S., Lamoreau B., Mohammad M., Barve S., McClain C., Joshi-Barve S. (2015). Molecular mechanisms of acrolein toxicity: Relevance to human disease. Toxicol. Sci..

[8] Iqubal A., Najmi A.K., Md S., Alkreathy H.M., Ali J., Syed M.A., Haque S.E. (2023). Oral delivery of nerolidol alleviates cyclophosphamide-induced renal inflammation, apoptosis, and fibrosis via modulation of NF-κB/cleaved caspase-3/TGF-β signaling molecules. Drug Deliv..

[9] Ijaz M.U., Mustafa S., Batool R., Naz H., Ahmed H., Anwar H. (2022). Ameliorative effect of herbacetin against cyclophosphamide-induced nephrotoxicity in rats via attenuation of oxidative stress, inflammation, apoptosis and mitochondrial dysfunction. Hum. Exp. Toxicol..

[10] Page M.J., McKenzie J.E., Bossuyt P.M., Boutron I., Hoffmann T.C., Mulrow C.D., Shamseer L., Tetzlaff J.M., Akl E.A., Brennan S.E. (2021). The PRISMA 2020 statement: An updated guideline for reporting systematic reviews. BMJ.

[11] Hooijmans C.R., Rovers M.M., de Vries R.B.M., Leenaars M., Ritskes-Hoitinga M., Langendam M.W. (2014). SYRCLE’s risk of bias tool for animal studies. BMC Med. Res. Methodol..

[12] Percie du Sert N., Hurst V., Ahluwalia A., Alam S., Avey M.T., Baker M., Browne W.J., Clark A., Cuthill I.C., Dirnagl U. (2020). The ARRIVE guidelines 2.0: Updated guidelines for reporting animal research. PLoS Biol..

[13] Vesterinen H.M., Sena E.S., Egan K.J., Hirst T.C., Churolov L., Currie G.L., Antonic A., Howells D.W., Macleod M.R. (2014). Meta-analysis of data from animal studies: A practical guide. J. Neurosci. Methods.

[14] Jiang S., Zhang Z., Huang F., Yang Z., Yu F., Tang Y., Ding G. (2020). Protective effect of low molecular weight peptides from *Solenocera crassicornis* head against cyclophosphamide-induced nephrotoxicity in mice via the Keap1/Nrf2 pathway. Antioxidants.

[15] Kabir F., Marashi N.S., Goudarzi M., Khalili H., Garaei N., Naghdi S., Rahmani R., Soroush E., Shokoohi F., Tabary P.Z. (2025). Protective efficacy of protocatechuic acid against cyclophosphamide-induced nephrotoxicity in small white mice. J. Biochem. Mol. Toxicol..

[16] Kalantar M., Goudarzi M., Khodayar M.J., Babaei J., Foruozandeh H., Bakhtiari N., Alidadi H. (2016). Protective effects of the hydroalcoholic extract of *Capparis spinosa* L. against cyclophosphamide-induced nephrotoxicity in mice. Jundishapur J. Nat. Pharm. Prod..

[17] Kang X., Jing M., Zhang G., He L., Hong P., Deng C. (2019). The ameliorating effect of plasma protein from *Tachypleus tridentatus* on cyclophosphamide-induced acute kidney injury in mice. Mar. Drugs.

[18] Lin X., Yang F., Huang J., Jiang S., Tang Y., Li J. (2020). Ameliorate effect of pyrroloquinoline quinone against cyclophosphamide-induced nephrotoxicity by activating the Nrf2 pathway and inhibiting the NLRP3 pathway. Life Sci..

[19] Mahipal P., Pawar R.S. (2017). Nephroprotective effect of *Murraya koenigii* on cyclophosphamide induced nephrotoxicity in rats. Asian Pac. J. Trop. Med..

[20] Mahmood N., Rashid B., Abdulla S., Marouf B., Hamaamin K., Othman H. (2025). Effects of zofenopril and thymoquinone in cyclophosphamide-induced urotoxicity and nephrotoxicity in rats; the value of their anti-inflammatory and antioxidant properties. J. Inflamm. Res..

[21] Mohamed O.S., Abdel Baky N.A., Sayed-Ahmed M.M., Al-Najjar A.H. (2023). Lactoferrin alleviates cyclophosphamide induced-nephropathy through suppressing the orchestration between Wnt4/β-catenin and ERK1/2/NF-κB signaling and modulating Klotho and Nrf2/HO-1 pathway. Life Sci..

[22] Mohsin N., Akhtar M.S., Alkahtani S.A., Walbi I.A., Alhazmi Y., Alam M.N., Bhardwaj A. (2024). Nephroprotective effect of bergapten against cyclophosphamide-mediated renal stress, inflammation, and fibrosis in Wistar rats: Probable role of NF-κB and TGF-β1 signaling molecules. ACS Omega.

[23] Hamzeh M., Talebpour Amiri F.T., Yaghubi-Beklar S., Hosseinimehr S.J. (2018). Nephroprotective Effect of Cerium Oxide Nanoparticles on Cyclophosphamide-Induced Nephrotoxicity via Anti-Apoptotic and Antioxidant Properties in BALB/c Mice. Marmara Pharm. J..

[24] Raoof S., Rezaei S., Zargari M., Mirzaei M., Hosseinimehr S.J., Karimpour Malekshah A., Talebpour Amiri F. (2025). Sinapic Acid Attenuated Nephrotoxicity against Cyclophosphamide in Mice Model: A Histochemical, Immunohistochemical and Histopathological Evaluation. Iran. J. Basic. Med. Sci..

[25] Said E., Elkashef W.F., Abdelaziz R.R. (2016). Tranilast ameliorates cyclophosphamide-induced lung injury and nephrotoxicity. Can. J. Physiol. Pharmacol..

[26] Salama R.M., Nasr M.M., Abdelhakeem J.I., Roshdy O.K., ElGamal M.A. (2022). Alogliptin attenuates cyclophosphamide-induced nephrotoxicity: A novel therapeutic approach through modulating MAP3K/JNK/SMAD3 signaling cascade. Drug Chem. Toxicol..

[27] Sayed-Ahmed M.M. (2010). Progression of cyclophosphamide-induced acute renal metabolic damage in carnitine-depleted rat model. Clin. Exp. Nephrol..

[28] Sharma S., Sharma P., Kulurkar P., Singh D., Kumar D., Patial V. (2017). Iridoid glycosides fraction from *Picrorhiza kurroa* attenuates cyclophosphamide-induced renal toxicity and peripheral neuropathy via PPAR-γ mediated inhibition of inflammation and apoptosis. Phytomedicine.

[29] Tohamy A.F., Hussein S., Moussa I.M., Rizk H., Daghash S., Alsubki R.A., Mubarak A.S., Alshammari H.O., Al-Maary K.S., Hemeg H.A. (2021). Lucrative antioxidant effect of metformin against cyclophosphamide induced nephrotoxicity. Saudi J. Biol. Sci..

[30] Wasim M., Ali S., Haque S. (2025). Verbenone prevents cyclophosphamide-induced oxidative stress inflammation fibrosis and cellular changes in the kidneys of Swiss albino mice by targeting NF-κB/TGF-β signaling pathways. Iran. J. Basic. Med. Sci..

[31] Waz S., Heeba G.H., Hassanin S.O., Abdel-latif R.G. (2021). Nephroprotective effect of exogenous hydrogen sulfide donor against cyclophosphamide-induced toxicity is mediated by Nrf2/HO-1/NF-κB signaling pathway. Life Sci..

[32] Zhu L., Luo C., Ma C., Kong L., Huang Y., Yang W., Huang C., Jiang W., Yi J. (2022). Inhibition of the NF-κB pathway and ERK-mediated mitochondrial apoptotic pathway takes part in the mitigative effect of betulinic acid on inflammation and oxidative stress in cyclophosphamide-triggered renal damage of mice. Ecotoxicol. Environ. Saf..

[33] Abed T.S., Al-Shawi N.N. (2025). Protective effects of irigenin against cyclophosphamide-induced nephrotoxicity in male rats: Comparative study with vitamin E. Al-Rafidain J. Med. Sci..

[34] Ahmad A.M., Mohammed H.A., Faris T.M., Hassan A.S., Mohamed H.B., El Dosoky M.I., Aboubakr E.M. (2021). Nano-structured lipid carrier-based oral glutathione formulation mediates renoprotection against cyclophosphamide-induced nephrotoxicity, and improves oral bioavailability of glutathione confirmed through RP-HPLC micellar liquid chromatography. Molecules.

[35] Alabi Q.K., Akomolafe R.O., Omole J.G., Aturamu A., Ige M.S., Kayode O.O., Kajewole-Alabi D. (2021). Polyphenol-rich extract of *Ocimum gratissimum* leaves prevented toxic effects of cyclophosphamide on the kidney function of Wistar rats. BMC Complement. Med. Ther..

[36] Aladaileh S.H., Al-Swailmi F.K., Abukhalil M.H., Shalayel M.H. (2021). Renoprotective effect of formononetin against cyclophosphamide-induced oxidative stress and inflammation in rat kidney. J. Pharm. Res. Int..

[37] Alghamdi A., Alissa M., Alghamdi S.A., Alshehri M.A., Alsuwat M.A., Alghamdi A. (2024). Suppression of glomerular damage, inflammation, apoptosis, and oxidative stress of acute kidney injury induced by cyclophosphamide toxicity using resveratrol in rat models. Tissue Cell.

[38] Alshahrani S., Ali Thubab H.M., Ali Zaeri A.M., Anwer T., Ahmed R.A., Jali A.M., Qadri M., Nomier Y., Moni S.S., Alam M.F. (2022). The protective effects of sesamin against cyclophosphamide-induced nephrotoxicity through modulation of oxidative stress, inflammatory-cytokines and apoptosis in rats. Int. J. Mol. Sci..

[39] Ayhanci A., Günes S., Sahinturk V., Appak S., Uyar R., Cengiz M., Altuner Y., Yaman S. (2010). Seleno L-methionine acts on cyclophosphamide-induced kidney toxicity. Biol. Trace Elem. Res..

[40] Caglayan C., Temel Y., Kandemir F.M., Yildirim S., Kucukler S. (2018). Naringin protects against cyclophosphamide-induced hepatotoxicity and nephrotoxicity through modulation of oxidative stress, inflammation, apoptosis, autophagy, and DNA damage. Environ. Sci. Pollut. Res. Int..

[41] Cengiz M., Peker Cengiz B., Teixeira Andrade A., Ayhanci A. (2024). Protective effect of escin against kidney injury: Histopathological and biochemical evaluations. Curr. Issues Mol. Biol..

[42] Gabr S.A., Elsaed W.M., Eladl M.A., Ghoniem G.A., El-Sherbiny M., El-Bayoumi K.S., Abouhish H., Desouky A.M., Abdel-Aziz M.M., Eldesoqui M. (2023). Circulating microRNAs as novel biomarkers for measuring the potency of ginger extract against cyclophosphamide toxicity in rat renal tissues: Molecular and histopathological study. Eur. Rev. Med. Pharmacol. Sci..

[43] Cuce G., Esen H.H., Koc T., Canbaz H.T., Limandal C., Kalkan S., Gürbilek M. (2016). Vitamin E partially ameliorates cyclophosphamide-induced nephrotoxicity in rats. Prog. Nutr..

[44] El-Naggar S.A., Alm-Eldeen A.A., Germoush M.O., El-Boray K.F., Elgebaly H.A. (2015). Ameliorative effect of propolis against cyclophosphamide-induced toxicity in mice. Pharm. Biol..

[45] El-Shabrawy M., Mishriki A., Attia H., Aboulhoda B.E., Emam M., Wanas H. (2020). Protective effect of tolvaptan against cyclophosphamide-induced nephrotoxicity in rat models. Pharmacol. Res. Perspect..

[46] Fouad A.A., Abdel-Gaber S.A., Abdelghany M.I. (2021). Hesperidin opposes the negative impact of cyclophosphamide on mice kidneys. Drug Chem. Toxicol..

[47] Goudarzi M., Khodayar M.J., Hosseini Tabatabaei S.M.T., Ghaznavi H., Fatemi I., Mehrzadi S. (2017). Pretreatment with melatonin protects against cyclophosphamide-induced oxidative stress and renal damage in mice. Fundam. Clin. Pharmacol..

[48] Goudarzi M., Esmaeilizadeh M., Dolatshahi M., Kalantar H., Frouzandeh H., Kalantar M. (2017). Protective effect of *Elaeagnus angustifolia* L. fruit hydroalcoholic extract on cyclophosphamide-induced nephrotoxicity in mice. Shiraz E-Med. J..

[49] Gunes S., Ayhanci A., Sahinturk V., Altay D.U., Uyar R. (2017). Carvacrol attenuates cyclophosphamide-induced oxidative stress in rat kidney. Can. J. Physiol. Pharmacol..

[50] Gunes S., Sahinturk V., Uslu S., Ayhanci A., Kacar S., Uyar R. (2018). Protective effects of selenium on cyclophosphamide-induced oxidative stress and kidney injury. Biol. Trace Elem. Res..

[51] Rasoul E., Hajipour B., Majidi H., Soleimani H. (2013). Vitamin E ameliorates cyclophosphamide induced nephrotoxicity. Life Sci. J..

[52] Kerimoğlu G., Arıcı T., Bıyık A.F., Kulaber A., Türkmen Alemdar N., Demir S., Aliyazıcıoğlu Y., Yenilmez E. (2023). Protective potential of pterostilbene against cyclophosphamide-induced nephrotoxicity and cystitis in rats. Int. Urol. Nephrol..

[53] Mansour D.F., Salama A.A.A., Hegazy R.R., Omara E.A., Nada S.A. (2017). Whey protein isolate protects against cyclophosphamide-induced acute liver and kidney damage in rats. J. Appl. Pharm. Sci..

[54] Mombeini M.A., Kalantar H., Sadeghi E., Goudarzi M., Khalili H., Kalantar M. (2022). Protective effects of berberine as a natural antioxidant and anti-inflammatory agent against nephrotoxicity induced by cyclophosphamide in mice. Naunyn Schmiedebergs Arch. Pharmacol..

[55] Obaid A.A., Alsammak M.I., Fadhil M.S. (2022). The effect of vitamin E on the histological structure of kidney in rats treated with cyclophosphamide. Iraqi J. Vet. Sci..

[56] Rehman M.U., Tahir M., Ali F., Qamar W., Lateef A., Khan R., Quaiyoom A., Oday-O-Hamiza, Sultana S. (2012). Cyclophosphamide-induced nephrotoxicity, genotoxicity, and damage in kidney genomic DNA of Swiss albino mice: The protective effect of ellagic acid. Mol. Cell. Biochem..

[57] Seker U., Kavak D.E., Dokumaci F.Z., Kizildag S., Irtegun-Kandemir S. (2024). The nephroprotective effect of quercetin in cyclophosphamide-induced renal toxicity might be associated with MAPK/ERK and NF-κB signal modulation activity. Drug Chem. Toxicol..

[58] Temel Y., Kucukler S., Yıldırım S., Caglayan C., Kandemir F.M. (2020). Protective effect of chrysin on cyclophosphamide-induced hepatotoxicity and nephrotoxicity via the inhibition of oxidative stress, inflammation, and apoptosis. Naunyn Schmiedebergs Arch. Pharmacol..

[59] Zhang Y., Chang J., Gao H., Qu X., Zhai J., Tao L., Sun J., Song Y. (2021). Huaiqihuang (HQH) granule alleviates cyclophosphamide-induced nephrotoxicity via suppressing the MAPK/NF-κB pathway and NLRP3 inflammasome activation. Pharm. Biol..

[60] Abraham P., Rabi S. (2011). Protective effect of aminoguanidine against cyclophosphamide-induced oxidative stress and renal damage in rats. Redox Rep..

[61] Alaqeel N.K., Al-Hariri M.T. (2023). Naringenin ameliorates cyclophosphamide-induced nephrotoxicity in experimental model. Saudi J. Biol. Sci..

[62] ALHaithloul H.A.S., Alotaibi M.F., Bin-Jumah M., Elgebaly H., Mahmoud A.M. (2019). *Olea europaea* leaf extract up-regulates Nrf2/ARE/HO-1 signaling and attenuates cyclophosphamide-induced oxidative stress, inflammation and apoptosis in rat kidney. Biomed. Pharmacother..

[63] Cengiz M. (2018). Boric acid protects against cyclophosphamide-induced oxidative stress and renal damage in rats. Cell. Mol. Biol..

[64] Dobrek Ł., Skowron B., Baranowska A., Płoszaj K., Bądziul D., Thor P. (2017). The influence of oxazaphosphorine agents on kidney function in rats. Medicina.

[65] Jiang X., Ren Z., Zhao B., Zhou S., Ying X., Tang Y. (2020). Ameliorating effect of pentadecapeptide derived from *Cyclina sinensis* on cyclophosphamide-induced nephrotoxicity. Mar. Drugs.

[66] Inker L.A., Eneanya N.D., Coresh J., Tighiouart H., Wang D., Sang Y., Crews D.C., Doria A., Estrella M.M., Froissart M. (2021). New Creatinine- and Cystatin C-Based Equations to Estimate GFR without Race. N. Engl. J. Med..

[67] Kellum J.A., Romagnani P., Ashuntantang G., Ronco C., Zarbock A., Anders H.-J. (2021). Acute Kidney Injury. Nat. Rev. Dis. Primers.

[68] Ostermann M., Legrand M., Meersch M., Srisawat N., Zarbock A., Kellum J.A. (2024). Biomarkers in Acute Kidney Injury. Ann. Intensive Care.

[69] Griffin B.R., Faubel S., Edelstein C.L. (2019). Biomarkers of Drug-Induced Kidney Toxicity. Ther. Drug Monit..

[70] Sauer J.-M., Porter A.C. (2021). Qualification of Translational Safety Biomarkers. Exp. Biol. Med..

[71] Fanouriakis A., Kostopoulou M., Andersen J., Aringer M., Arnaud L., Bae S.-C., Boletis J., Bruce I.N., Cervera R., Doria A. (2024). EULAR Recommendations for the Management of Systemic Lupus Erythematosus: 2023 Update. Ann. Rheum. Dis..

[72] Renaghan A.D., Ostermann M., Ronco C., Ballen K., Cosmai L., Fenoglio R., Floris M., Forni L.G., Gladstone D.E., Glezerman I.G. (2025). The Nephrotoxic Effects of Anti-Cancer Therapies: Consensus Report of the 34th Acute Disease Quality Initiative Workgroup. Nat. Rev. Nephrol..

[73] Raiola A.M., Bruno B., Risitano A.M., Mosna F., Cavattoni I.M., Onida F., Saporiti G., Patriarca F., Battista M.L., Pavone V. (2025). Posttransplant Cyclophosphamide as GVHD Prophylaxis in Patients Receiving Mismatched Unrelated HCT: The PHYLOS Trial. Blood Adv..

