# Supplementary material for: Cyclophosphamide-Induced Nephrotoxicity and Nephroprotection in Rodent Models: A Systematic Review and Random-Effects Meta-Analysis (2010–2025)
Source: J Xenobiot. 2026 Mar 4;16(2):48. doi: 10.3390/jox16020048 (PMC13010739; doi:10.3390/jox16020048)
Supplement: Supplementary file 1 [file jox-16-00048-s001.zip › jox-4158468-supplementary.pdf]

Journal of *Xenobiotics*

MDPI

Supplementary Materials: Cyclophosphamide-Induced Nephrotoxicity and Nephroprotection in Rodent Models: A Systematic Review and Random-Effects Meta-Analysis (2010–2025)

Denis Oberiukhtin, Anton Chernitskiy, Desheng Hu and Alexey Sarapultsev

Table S1. Evidence base size per endpoint and contrast

Outcome	Contrast	Metric	k	Studies	Unique_timepoints
Serum creatinine	CP vs control	MD (mg/dL)	11	11	11
Serum creatinine	Intervention+CP vs CP	MD (mg/dL)	14	10	11
Serum urea	CP vs control	MD (mg/dL)	11	11	11
Serum urea	Intervention+CP vs CP	MD (mg/dL)	14	10	11
Kidney MDA	CP vs control	Hedges' g (SMD)	6	6	6
Kidney MDA	Intervention+CP vs CP	Hedges' g (SMD)	10	6	10
Kidney GSH	CP vs control	Hedges' g (SMD)	7	7	7
Kidney GSH	Intervention+CP vs CP	Hedges' g (SMD)	11	7	11

Table S2. Tabulated statistics vs figure-only/missing for core outcomes (study × outcome accounting)

Outcome_name	studies_with_tabulated_stats	total_studies	studies_figure_only_or_missing_stats
Creatinine_serum	11	15	4
Urea_serum	11	15	4
BUN_serum	2	6	4
MDA_kidney	6	13	7
GSH_kidney	7	13	6

Table S3. Studies with figure-only or non-extractable reporting for at least one core outcome under the no-digitisation rule (listed as study IDs).

StudyID
ALHaithloul_2019_OleaLeaf
AbrahamRabi_2011
Ahmad_2021_GSHNLC
Alghamdi_2024_Resveratrol
Fouad_2019_Hesperidin
Gabr_2023_Nerolidol
Ijaz_2022_Ginger
Mansour_2017_Silymarin
Mohsin_2024_Bergapten
Mombeini_2022_Berberine
Rehman_2012_EllagicAcid

Table S4. Egger's regression (performed only when k ≥ 10)

Outcome	Contrast	k	Intercept	p
Serum creatinine	CP vs control	11	19.533	0.109
Serum creatinine	Intervention+CP vs CP	14	-14.713	0.0342
Serum urea	CP vs control	11	17.550	0.338
Serum urea	Intervention+CP vs CP	14	-9.150	0.266
Kidney MDA	Intervention+CP vs CP	10	-4.571	2.74e-05
Kidney GSH	Intervention+CP vs CP	11	4.770	6.19e-08

https://doi.org/10.3390/10.3390/jox16020048

Table S5. Full electronic search strategies and limits.

Database / platform	Interface / field tags	Search string	Limits / filters	Last search date	Records retrieved (n)
PubMed (MEDLINE)	Title/Abstract; Date - Publication	("cyclophosphamide-induced nephrotoxicity"[Title/Abstract] OR "cyclophosphamide-induced kidney injury"[Title/Abstract] OR "cyclophosphamide-induced renal injury"[Title/Abstract] OR ("cyclophosphamide"[Title/Abstract] AND ("nephrotoxicity"[Title/Abstract] OR "renal toxicity"[Title/Abstract] OR "kidney injury"[Title/Abstract]))) AND ("oxidative stress"[Title/Abstract] OR "inflammation"[Title/Abstract] OR "apoptosis"[Title/Abstract] OR "fibrosis"[Title/Abstract]) AND ("rat"[Title/Abstract] OR "rats"[Title/Abstract] OR "mouse"[Title/Abstract] OR "mice"[Title/Abstract] OR "animal model"[Title/Abstract] OR "in vivo"[Title/Abstract]) NOT ("liver"[Title/Abstract] OR "testis"[Title/Abstract] OR "ovary"[Title/Abstract] OR "lung"[Title/Abstract] OR "heart"[Title/Abstract] OR "brain"[Title/Abstract]) AND ("2010/01/01"[Date - Publication] : "2025/12/31"[Date - Publication]) 57 articles (20.11.2025) All search results should be exported to Zotero for deduplication and screening.	2010/01/01–2025/12/31; English or Russian; excluded non-renal organs (liver/testis/ovary/lung/heart/brain)	25/11/2025	59
Scopus (Elsevier)	TITLE-ABS-KEY / TITLE-ABS	Timeframe: 2010 – 2025. Search string (PubMed): ("cyclophosphamide-induced nephrotoxicity"[Title/Abstract] OR "cyclophosphamide-induced kidney injury"[Title/Abstract] OR "cyclophosphamide-induced renal injury"[Title/Abstract]	PUBYEAR 2010–2025; DOCTYPE=ar; LANGUAGE=English or Russian; organ	25/11/2025	32

**Database / platform**	**Interface / field tags**	**Search string**	**Limits / filters**	**Last search date**	**Records retrieved (n)**
		OR ("cyclophosphamide"[Title/Abstract] AND ("nephrotoxicity"[Title/Abstract] OR "renal toxicity"[Title/Abstract] OR "kidney injury"[Title/Abstract]))) AND ("oxidative stress"[Title/Abstract] OR "inflammation"[Title/Abstract] OR "apoptosis"[Title/Abstract] OR "fibrosis"[Title/Abstract]) AND ("rat"[Title/Abstract] OR "rats"[Title/Abstract] OR "mouse"[Title/Abstract] OR "mice"[Title/Abstract] OR "animal model"[Title/Abstract] OR "in vivo"[Title/Abstract]) NOT ("liver"[Title/Abstract] OR "testis"[Title/Abstract] OR "ovary"[Title/Abstract] OR "lung"[Title/Abstract] OR "heart"[Title/Abstract] OR "brain"[Title/Abstract]) AND ("2010/01/01"[Date - Publication] : "2025/12/31"[Date - Publication]) 57 articles (20.11.2025) All search results should be exported to Zotero for deduplication and screening. 1. Scopus (Elsevier) TITLE-ABS (("cyclophosphamide-induced nephrotoxicity" OR "cyclophosphamide-induced kidney injury" OR "cyclophosphamide-induced renal injury" OR ("cyclophosphamide" AND ("nephrotoxicity" OR "renal toxicity" OR "kidney injury"))) AND ("oxidative stress" OR "inflammation" OR "apoptosis" OR "fibrosis") AND (rat OR rats OR mouse OR mice OR "animal model" OR "in vivo")) AND NOT TITLE-ABS (liver OR testis OR ovary OR lung OR heart OR brain) AND (PUBYEAR > 2009 AND PUBYEAR < 2026) AND (LIMIT-TO	exclusions via NOT TITLE-ABS		

Database / platform	Interface / field tags	Search string	Limits / filters	Last search date	Records retrieved (n)
		(DOCTYPE , "ar")) AND (LIMIT-TO (LANGUAGE , "English") OR LIMIT-TO (LANGUAGE , "Russian")) 4.			
Web of Science Core Collection	TS= (topic search)	(<br>TS=(<br>cyclophosphamide<br>AND<br>(nephrotoxic* OR nephrotoxicit* OR nephropath* OR "kidney injury" OR "renal injury" OR "acute kidney injury" OR AKI OR "renal dysfunction" OR "kidney damage")<br>AND<br>(rat OR rats OR mouse OR mice OR murine OR rodent* OR Wistar OR "Sprague Dawley" OR BALB/c OR "C57BL/6")<br>)<br>NOT<br>TS=(liver OR hepatic OR testis OR ovarian OR ovary OR lung OR cardiac OR heart OR brain)<br>)<br>AND PY=2010-2025<br>AND (LA=(English OR Russian))<br>AND DT=(Article)	2010–2025; English or Russian; document type: Article	25 Nov 2025	
Embase (Elsevier)	Emtree /exp + ti,ab	('cyclophosphamide'/exp OR 'cyclophosphamide':ab,ti) AND ('nephrotoxicity'/exp OR 'kidney injury'/exp OR 'renal toxicity':ab,ti) AND ('oxidative stress'/exp OR 'inflammation'/exp OR 'apoptosis'/exp OR 'fibrosis'/exp) AND ('rat'/exp OR 'mouse'/exp OR 'animal model':ab,ti	Animal experiment; 2010–2025; English or Russian; organ exclusions	25/11/2025	32

Database / platform	Interface / field tags	Search string	Limits / filters	Last search date	Records retrieved (n)
		OR 'in vivo':ab,ti) NOT ('liver'/exp OR 'testis'/exp OR 'ovary'/exp OR 'lung'/exp OR 'heart'/exp OR 'brain'/exp) AND [2010-2025]/py 5.			
Google Scholar (scoping)	Free text	"cyclophosphamide-induced nephrotoxicity" OR "cyclophosphamide-induced kidney injury" "oxidative stress" OR "inflammation" OR "apoptosis" OR "fibrosis" rat OR mouse OR "animal model" OR "in vivo" -"liver" -"testis" -"ovary" -"heart" -"lung" -"brain" 2010..2025	2010–2025; English/Russian; organ exclusions with minus terms	25 Nov 2025	25

Table S6. Study-level coverage of outcome domains in cyclophosphamide-induced nephrotoxicity models (mapping matrix across included interventions)

Ref. #	Species	Model type	Timepoint	Intervention	F	OX	INF	APO	FIB	BIO	HIST
8	Mouse	ac_single	24h_post_CP	Nerolidol	✓	✓	✓	✓	✓	✓	✓
9	Rat	ac_single	24h_post_CP	Herbacetin	✓	✓	✓	✓	—	✓	✓
14	Mouse	subacute_repeated	after_last_CP	SCP_LMWP (Jiang S., 2020)	✓	✓	—	—	—	—	—
15	Mouse	subacute_repeated	after_last_CP	Protocatechuic_acid	✓	✓	✓	—	—	✓	✓
16	Rat	ac_single	24h_post_CP	Capparis_spinosa_extract	✓	✓	—	—	—	—	✓
17	Mouse	unknown	NR	Tachypleus_tridentatus_plasma_protein	✓	—	✓	✓	—	—	✓
18	Rat	ac_single	72h_post_CP	Pyrroloquinoline_quinone	✓	✓	✓	—	—	—	✓
19	Rat	ac_single	24h_post_CP	Murraya_koenigii_extract	✓	✓	—	—	—	—	✓
20	Rat	ac_single	72h_post_CP	Thymoquinone / Zofenopril / Zofenopril+Thymoquinone	✓	✓	✓	—	✓	✓	✓
21	Mouse	ac_single	72h_post_CP	Lactoferrin	✓	✓	✓	✓	✓	—	✓
22	Mouse	ac_single	72h_post_CP	Bergapten	✓	✓	✓	—	✓	✓	✓
23	Rat	ac_single	24h_post_CP	Cerium_oxide_nanoparticles	✓	✓	—	✓	—	—	✓
24	Mouse	ac_single	day10_per_protocol	Sinapic_acid	✓	✓	✓	✓	—	—	✓
25	Rat	ac_single	24h_post_CP	Tranilast	✓	✓	✓	—	—	—	✓
26	Rat	ac_single	72h_post_CP	Alogliptin	✓	✓	✓	✓	✓	✓	✓
27	Rat	ac_single	24h_post_CP	Propionyl-L-carnitine	✓	✓	—	—	—	—	—
28	Rat	ac_single	24h_post_CP	Picrorhiza_iridoid_fraction	✓	✓	✓	✓	—	—	✓

https://doi.org/10.3390/10.3390/jox16020048

29	Rat	ac_single	72h_post_CP	Metformin	✓	✓	—	✓	—	—	✓
30	Rat	ac_single	72h_post_CP	Verbenone	✓	✓	✓	—	✓	—	✓
31	Rat	ac_single	72h_post_CP	H2S_donor	✓	✓	✓	✓	—	—	✓
32	Mouse	ac_single	72h_post_CP	Betulinic_acid	✓	✓	✓	✓	✓	✓	✓
33	Rat	ac_single	24h_post_CP	Irigenin / Vitamin_E	✓	✓	—	—	—	—	✓
34	Rat	ac_single	Time_from_paper	GSH_NLCs / Glutathione_free	✓	✓	✓	✓	—	—	✓
35	Rat	subacute_repeated	day8	Ocimum_gratissimum	✓	✓	✓	—	—	—	✓
36	Rat	ac_single	24h_post_CP	Formononetin	✓	✓	✓	✓	—	—	—
37	Rat	ac_single	24h_post_CP	Resveratrol	✓	✓	✓	—	—	—	✓
38	Rat	ac_single	Time_from_paper	Sesamin	✓	✓	✓	✓	—	—	✓
39	Rat	ac_single	24h_post_last_CP	Seleno_L_methionine	—	✓	—	—	—	—	✓
40	Rat	ac_single	Time_from_paper	Naringin	✓	✓	✓	✓	—	—	✓
41	Rat	ac_single	Time_from_figure	Escin	✓	—	—	✓	✓	—	✓
42	Rat	atypical_repeated	Time_from_paper	Ginger_extract	—	✓	—	✓	✓	—	✓
43	Rat	subacute_repeated	Time_from_paper	Vitamin_E	✓	✓	—	✓	—	—	✓
44	Mouse	ac_single	Time_from_paper	Propolis	✓	✓	—	—	—	—	—
45	Rat	subacute_repeated	Time_from_paper	Tolvaptan	✓	✓	—	—	—	—	—
46	Mouse	ac_single	Time_from_paper	Hesperidin	✓	✓	✓	✓	—	✓	✓
47	Mouse	ac_single	24h_post_CP	Melatonin (Goudarzi 2017; Fundam Clin Pharmacol)	✓	✓	—	—	—	—	—
48	Mouse	ac_single	24h_post_CP	Elaeagnus_angustifolia (Goudarzi 2017; Shiraz E-Med J)	✓	✓	—	—	—	—	✓
49	Rat	ac_single	24h_post_CP	Carvacrol	✓	✓	—	—	—	—	✓
50	Rat	ac_single	24h_post_CP	Selenium	✓	✓	—	—	—	—	✓
51	Rat	ac_single	Time_from_paper	Vitamin_E	✓	✓	—	—	—	—	—
52	Rat	ac_single	24h_post_CP	Pterostilbene	✓	✓	—	—	—	—	—
53	Rat	ac_single	24h_post_CP	Whey_protein_isolate	✓	✓	✓	—	—	—	✓
54	Rat	ac_single	24h_post_CP	Berberine	✓	✓	✓	—	✓	✓	✓
55	Rat	subacute_repeated	after_last_CP	Vitamin_E	✓	—	—	—	—	—	✓
56	Mouse	unknown	NR	Ellagic_acid	✓	✓	—	—	—	—	✓
57	Rat	ac_single	72h_post_CP	Quercetin	✓	✓	✓	✓	—	—	✓
58	Rat	ac_single	24h_post_CP	Chrysin	✓	✓	✓	✓	—	—	✓
59	Mouse	ac_single	72h_post_CP	Huaiqihuang_granule	✓	✓	✓	—	—	—	✓

https://doi.org/10.3390/10.3390/jox16020048

60	Rat	ac_single	16h_post_CP	Aminoguanidine	—	✓	—	—	—	—	—
61	Rat	subacute_repeated	end_of_protocol_day21	Naringenin	✓	✓	—	—	—	✓	✓
62	Rat	ac_single	72h_post_CP	Olea_europaea_leaf_extract	✓	✓	✓	✓	—	—	✓
63	Rat	ac_single	Time_from_paper	Boric_acid	—	✓	✓	—	—	—	✓
64	Rat	ac_single	Time_from_paper	Oxazaphosphorines (CP vs IF)	✓	✓	—	—	✓	✓	✓
65	Mouse	subacute_repeated	after_last_CP	Cyclina_peptide (Jiang X., 2020)	✓	✓	✓	✓	✓	✓	✓

Notes: F, functional renal markers (e.g., serum creatinine [Cr], urea/BUN); OX, oxidative stress markers (e.g., MDA/TBARS, GSH, antioxidant enzymes); INF, inflammatory markers (e.g., TNF/ILs, NF-κB, MPO, NLRP3, MAPK); APO, apoptosis markers (e.g., caspases, Bax/Bcl-2, cytochrome c, TUNEL); FIB, fibrosis/remodelling markers (e.g., TGF-β, Smad, collagen/ECM, α-SMA, Wnt/β-catenin); BIO, novel kidney injury biomarkers (e.g., KIM-1, NGAL, cystatin C); HIST, histopathology (e.g., H&E, PAS, Masson's trichrome, semi-quantitative scoring); ✓ indicates the domain was assessed; — indicates not reported/assessed; NR, not reported.

Table S7. Audit trail for quantitative synthesis (StudyID-to-reference mapping and pooled strata). This table documents (i) the StudyID nomenclature used in the quantitative synthesis and its mapping to the reference numbering in the main manuscript, and (ii) the composition of each meta-analytic pool by outcome and contrast. Excluded studies and reasons are listed at the end.

A) StudyID-to-reference mapping (RefNo corresponds to the reference number used in Table 1 and the main reference list).

**StudyID**	**RefNo**	**Citation (short)**
Mohsin_2024_Bergapten	22	Mohsin (2024.0), Bergapten
AbedAlShawi_2025	33	Abed (2025.0), Irigenin; Vitamin_E
Ahmad_2021_GSHNLC	34	Ahmad (2021.0), GSH_NLCs; Glutathione_free
Alabi_2021	35	Alabi (2021.0), Ocimum_gratissimum
Aladaileh_2021	36	Aladaileh (2021.0), Formononetin
Alghamdi_2024_Resveratrol	37	Alghamdi (2024.0), Resveratrol
Alshahrani_2022_Sesamin	38	Alshahrani (2022.0), Sesamin
Ayhanci_2010_SLM	39	Ayhanci (2010.0), Seleno_L_methionine
Caglayan_2018_Naringin	40	Caglayan (2018.0), Naringin
Cengiz_2024_Escin	41	Cengiz (2024.0), Escin
Gabr_2023_Ginger_miRNAs	42	Gabr (2023.0), Ginger_extract

StudyID	RefNo	Citation (short)
Cuce_2016_VitE	43	Cuce (2016.0), Vitamin_E
ElNaggar_2015_Propolis	44	El-Naggar (2015.0), Propolis
ElShabrawy_2020_Tolvaptan	45	El-Shabrawy (2020.0), Tolvaptan
Fouad_2019_Hesperidin	46	Fouad (2021.0), Hesperidin
Goudarzi_2017_Melatonin	47	Goudarzi (2017.0), Melatonin
Gunes_2017_Carvacrol	49	Gunes (2017.0), Carvacrol
Gunes_2018_Selenium	50	Gunes (2018.0), Selenium
Mombeini_2022_Berberine	54	Mombeini (2022.0), Berberine
AbrahamRabi_2011	60	Abraham (2011.0), Aminoguanidine
Alaqeel_2023_Naringenin	61	Alaqeel (2023.0), Naringenin
ALHaithloul_2019_OleaLeaf	62	ALHaithloul (2019.0), Olea_europaea_leaf_extract
Cengiz_2018_BoricAcid	63	Cengiz (2018.0), Boric_acid
Dobrek_2017_CP	64	Dobrek (2017.0), nan

B) Meta-analytic pools (as implemented) and number of effect sizes/studies.

Outcome	Contrast	k (effect sizes)	Studies	Unique timepoints
Creatinine_serum	CP_vs_Control	9	9	9
Creatinine_serum	IntCP_vs_CP	11	8	8
Urea_serum	CP_vs_Control	9	9	9
Urea_serum	IntCP_vs_CP	11	8	8
MDA_kidney	CP_vs_Control	6	6	6
MDA_kidney	IntCP_vs_CP	10	6	6
GSH_kidney	CP_vs_Control	7	7	7
GSH_kidney	IntCP_vs_CP	11	7	7

C) Studies excluded from quantitative pooling under the no-digitisation rule (core outcomes in figures only).

StudyID	Reason
ALHaithloul_2019_OleaLeaf	Core outcomes reported in figures only (no extractable n/mean/SD); excluded from meta-analysis under the no-digitisation rule; retained for narrative synthesis.
AbrahamRabi_2011	Core outcomes reported in figures only (no extractable n/mean/SD); excluded from meta-analysis under the no-digitisation rule; retained for narrative synthesis.
Ahmad_2021_GSHNLC	Core outcomes reported in figures only (no extractable n/mean/SD); excluded from meta-analysis under the no-digitisation rule; retained for narrative synthesis.
Alghamdi_2024_Resveratrol	Core outcomes reported in figures only (no extractable n/mean/SD); excluded from meta-analysis under the no-digitisation rule; retained for narrative synthesis.
Fouad_2019_Hesperidin	Core outcomes reported in figures only (no extractable n/mean/SD); excluded from meta-analysis under the no-digitisation rule; retained for narrative synthesis.
Gabr_2023_Ginger_miRNAs	Core outcomes reported in figures only (no extractable n/mean/SD); excluded from meta-analysis under the no-digitisation rule; retained for narrative synthesis.
Goudarzi_2017_Melatonin	Core outcomes reported in figures only (no extractable n/mean/SD); excluded from meta-analysis under the no-digitisation rule; retained for narrative synthesis.
Gunes_2017_Carvacrol	Core outcomes reported in figures only (no extractable n/mean/SD); excluded from meta-analysis under the no-digitisation rule; retained for narrative synthesis.
Gunes_2018_Selenium	Core outcomes reported in figures only (no extractable n/mean/SD); excluded from meta-analysis under the no-digitisation rule; retained for narrative synthesis.
Mohsin_2024_Bergapten	Core outcomes reported in figures only (no extractable n/mean/SD); excluded from meta-analysis under the no-digitisation rule; retained for narrative synthesis.
Mombeini_2022_Berberine	Core outcomes reported in figures only (no extractable n/mean/SD); excluded from meta-analysis under the no-digitisation rule; retained for narrative synthesis.

Table S8. Study-level SYRCLE risk-of-bias judgements (domain-by-domain) Judgements are reported as Low / High / Unclear risk of bias per SYRCLE domains.

RefNo	Study	SG	BC	AC	RH	BL_CI	RA	BL_OA	IOD	SOR	OB
8	Iqubal 2023	Unclear	Unclear	Unclear	Unclear	Unclear	Unclear	Unclear	Unclear	Unclear	Unclear
9	Ijaz 2022	Unclear	Unclear	Unclear	Unclear	Unclear	Unclear	Unclear	Unclear	Unclear	Unclear
14	Jiang S. 2020 (Antioxidants)	Unclear	Unclear	Unclear	Unclear	Unclear	Unclear	Unclear	Low	Unclear	Unclear

https://doi.org/10.3390/10.3390/jox16020048

RefNo	Study	SG	BC	AC	RH	BL_CI	RA	BL_OA	IOD	SOR	OB
15	Kabir 2025	Unclear	Unclear	Unclear	Unclear	Unclear	Unclear	Unclear	Unclear	Unclear	Unclear
16	Kalantar 2016	High	Unclear	Unclear	Unclear	Unclear	Unclear	Unclear	Unclear	Unclear	Unclear
17	Kang 2019	High	Unclear	Unclear	Unclear	Unclear	Unclear	Unclear	Unclear	Unclear	Unclear
18	Lin 2020	Low	Unclear	Unclear	Unclear	Unclear	Unclear	Unclear	Unclear	Unclear	Unclear
19	Mahipal 2017	Unclear	Unclear	Unclear	Unclear	Unclear	Unclear	Unclear	Unclear	Unclear	Unclear
20	Mahmood 2025	High	Unclear	Unclear	Unclear	Unclear	Unclear	Unclear	Unclear	Unclear	Unclear
21	Mohamed 2023	Unclear	Unclear	Unclear	Unclear	Unclear	Unclear	Low	Unclear	Unclear	Unclear
22	Mohsin 2024	High	Unclear	Unclear	Unclear	Unclear	Unclear	Unclear	Unclear	Unclear	Unclear
23	Hamzeh 2018	Unclear	Unclear	Unclear	Unclear	Unclear	Unclear	Low	Unclear	Unclear	Unclear
24	Raoof 2025	Unclear	Unclear	Unclear	Unclear	Unclear	Unclear	Low	Unclear	Unclear	Unclear
25	Said 2016	Unclear	Unclear	Unclear	Unclear	Unclear	Unclear	Low	Unclear	Unclear	Unclear
26	Salama 2022	Unclear	Unclear	Unclear	Unclear	Low	Unclear	Low	Unclear	Unclear	Unclear
27	Sayed-Ahmed 2010	Low	Unclear	Unclear	Unclear	Unclear	Unclear	Low	Unclear	Unclear	Unclear
28	Sharma 2017	High	Unclear	Unclear	Unclear	Unclear	Unclear	Unclear	Unclear	Unclear	Unclear
29	Tohamy 2021	Unclear	Unclear	Unclear	Unclear	Unclear	Unclear	Unclear	Unclear	Unclear	Unclear
30	Wasim 2025	High	Unclear	Unclear	Unclear	Unclear	Unclear	Unclear	Unclear	Unclear	Unclear
31	Waz 2021	Unclear	Unclear	Unclear	Unclear	Unclear	Unclear	Unclear	Unclear	Unclear	Unclear
32	Zhu 2022	High	Unclear	Unclear	Unclear	Unclear	Unclear	Unclear	Unclear	Unclear	High
33	Abed 2025	Unclear	Unclear	Unclear	Unclear	Unclear	Unclear	Unclear	Unclear	Unclear	Unclear
34	Ahmad 2021	Unclear	Unclear	Unclear	Unclear	Unclear	Unclear	Unclear	Unclear	Unclear	Unclear
35	Alabi 2021	Unclear	Unclear	Unclear	Unclear	Unclear	Unclear	Low	Unclear	Unclear	Unclear
36	Aladaileh 2021	Unclear	Unclear	Unclear	Unclear	Unclear	Unclear	Unclear	Unclear	Unclear	Unclear
37	Alghamdi 2024	Unclear	Unclear	Unclear	Unclear	Unclear	Unclear	Unclear	Unclear	Unclear	Unclear
38	Alshahrani 2022	Unclear	Unclear	Unclear	Unclear	Unclear	Unclear	Unclear	Unclear	Unclear	Unclear
39	Ayhanci 2010	Unclear	Unclear	Unclear	Unclear	Unclear	Unclear	Unclear	Unclear	Unclear	Unclear
40	Caglayan 2018	Unclear	Unclear	Unclear	Unclear	Unclear	Unclear	Unclear	Unclear	Unclear	Unclear
41	Cengiz 2024	Unclear	Unclear	Unclear	Unclear	Unclear	Unclear	Low	Unclear	Unclear	Unclear
42	Gabr 2023	Unclear	Unclear	Unclear	Unclear	Unclear	Unclear	Unclear	Unclear	Unclear	Unclear
43	Cuce 2016	High	Unclear	Unclear	Unclear	Unclear	Unclear	Unclear	Unclear	Unclear	Unclear
44	El-Naggar 2015	Unclear	Unclear	Unclear	Unclear	Unclear	Unclear	Unclear	Unclear	Unclear	Unclear
45	El-Shabrawy 2020	Unclear	Unclear	Unclear	Unclear	Unclear	Unclear	Unclear	Unclear	Unclear	Unclear
46	Fouad 2021	Low	Unclear	Unclear	Unclear	Unclear	Unclear	Low	Unclear	Unclear	Unclear

https://doi.org/10.3390/10.3390/jox16020048

RefNo	Study	SG	BC	AC	RH	BL_CI	RA	BL_OA	IOD	SOR	OB
47	Goudarzi 2017 (melatonin)	Unclear	Unclear	Unclear	Unclear	Unclear	Unclear	Low	Unclear	Unclear	Unclear
48	Goudarzi 2017 (Elaeagnus)	High	Unclear	Unclear	Unclear	Unclear	Unclear	Low	Unclear	Unclear	Unclear
49	Gunes 2017	Unclear	Unclear	Unclear	Unclear	Unclear	Unclear	Unclear	Unclear	Unclear	Unclear
50	Gunes 2018	Unclear	Unclear	Unclear	Unclear	Unclear	Unclear	Unclear	Unclear	Unclear	Unclear
51	Rasoul 2013	Unclear	Unclear	Unclear	Unclear	Unclear	Unclear	Unclear	Unclear	Unclear	Unclear
52	Kerimoğlu 2023	Unclear	Unclear	Unclear	Unclear	Unclear	Unclear	Low	Unclear	Unclear	Unclear
53	Mansour 2017	Unclear	Unclear	Unclear	Unclear	Unclear	Unclear	Unclear	Unclear	Unclear	Unclear
54	Mombeini 2022	Unclear	Unclear	Unclear	Unclear	Unclear	Unclear	Low	Unclear	Unclear	Unclear
55	Obaid 2022	Unclear	Unclear	Unclear	Unclear	Unclear	Unclear	Unclear	Unclear	Unclear	Unclear
56	Rehman 2012	High	Unclear	Unclear	Unclear	Unclear	Unclear	Unclear	Unclear	Unclear	Unclear
57	Seker 2024	High	Unclear	Unclear	Unclear	Unclear	Unclear	Unclear	Unclear	Unclear	Unclear
58	Temel 2020	Unclear	Unclear	Unclear	Unclear	Unclear	Unclear	Unclear	Unclear	Unclear	Unclear
59	Zhang 2021	Unclear	Unclear	Unclear	Unclear	Unclear	Unclear	Low	Unclear	Unclear	High
60	Abraham 2011	Unclear	Unclear	Unclear	Unclear	Unclear	Unclear	Unclear	High	Unclear	Unclear
61	Alaqeel 2023	Unclear	Unclear	Unclear	Unclear	Unclear	Unclear	Unclear	Unclear	Unclear	Unclear
62	ALHaithloul 2019	Unclear	Unclear	Unclear	Unclear	Unclear	Unclear	Unclear	Unclear	Unclear	Unclear
63	Cengiz 2018	Unclear	Unclear	Unclear	Unclear	Unclear	Unclear	Unclear	Unclear	Unclear	Unclear
64	Dobrek 2017	Unclear	Low	Unclear	Unclear	Unclear	Unclear	Unclear	Low	Unclear	Unclear
65	Jiang X. 2020 (Marine Drugs)	Unclear	Unclear	Unclear	Unclear	Unclear	Unclear	Unclear	Unclear	Unclear	Unclear

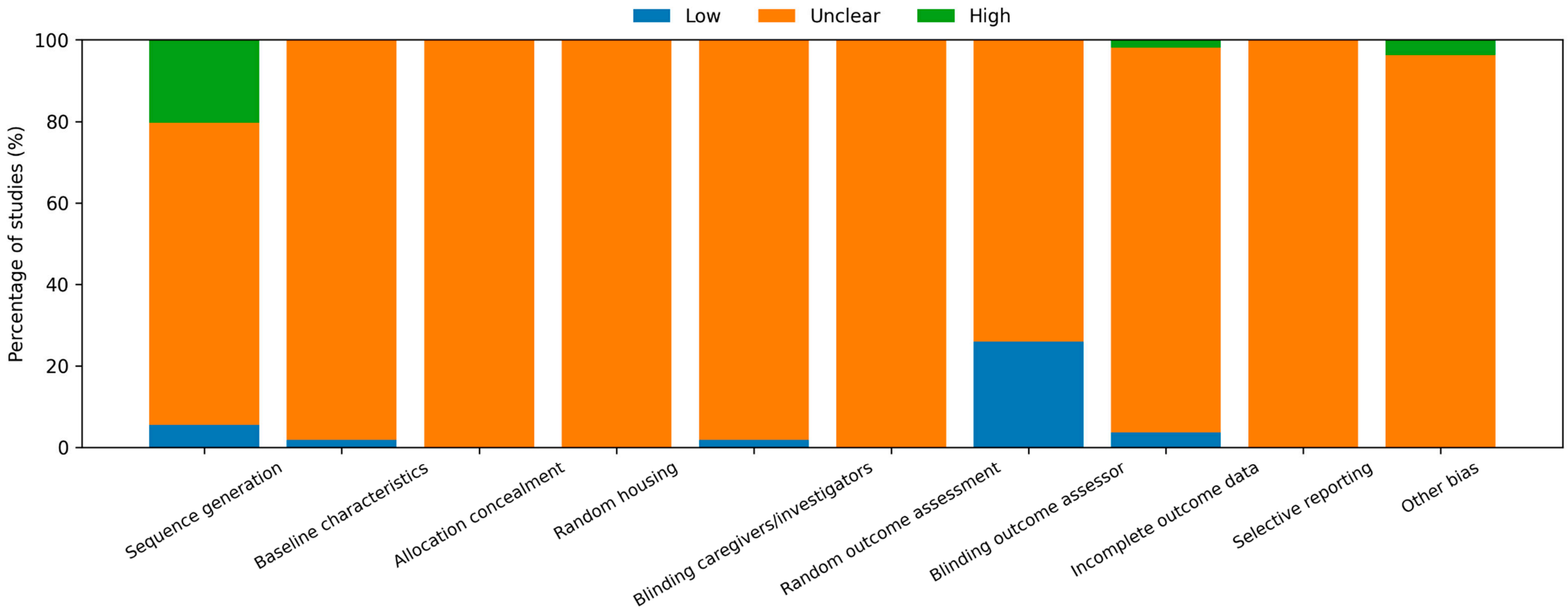

Figure S1. Study-level SYRCLE risk-of-bias profile across included studies (n = 54).

Stacked bar chart showing, for each SYRCLE domain, the percentage of included studies judged at low, unclear, or high risk of bias. Domains comprise sequence generation, baseline characteristics, allocation concealment, random housing, blinding of caregivers/investigators, random outcome assessment, blinding of outcome assessors, incomplete outcome data, selective outcome reporting, and other sources of bias. Judgements were assigned at the study level based on explicit reporting in the original publications; "unclear" indicates insufficient methodological detail to support a low- or high-risk judgement
